# MRAS: Master Regulator Analysis of Alternative Splicing

**DOI:** 10.1002/advs.202414493

**Published:** 2025-05-05

**Authors:** Lei Zhou, Yue Huang, Yang Zhao, Dan Guo, Xiao Wen, Ruihong Xu, Xuan Lv, Song Wu, Sicheng Jing, Zhaoqi Liu

**Affiliations:** ^1^ China National Center for Bioinformation Beijing 100101 China; ^2^ Beijing Institute of Genomics Chinese Academy of Sciences Beijing 100101 China; ^3^ University of Chinese Academy of Sciences Beijing 100049 China; ^4^ National Genomics Data Center China National Center for Bioinformation Beijing 100101 China; ^5^ Department of Biology University of California San Diego San Diego CA 92122 USA

**Keywords:** alternative splicing, master regulator analysis, RNA binding protein, splicing regulatory network

## Abstract

As a molecular feature that characterizes most tumor types, cancer‐associated splicing dysregulation largely arises from recurrent genetic mutations and altered expression of *trans*‐acting splicing factors. Although splicing factor mutations occur less frequently in solid tumors, splicing disorders are pervasive and proven to promote tumorigenesis. However, it still lacks an efficient way to identify the key splicing factors at the top regulatory hierarchy whose abnormal expressions induce such splicing disorders and drive phenotypic variability. Here, MRAS (Master Regulator analysis of Alternative Splicing) is introduced, a computational method designed to pinpoint the pivotal splicing factors that play a central role in shaping splicing regulatory networks and influencing cellular processes. MRAS is demonstrated its power by identifying master splicing regulators associated with various disease phenotypes, including tumor initiation, progression, metastasis, and treatment resistance. Moreover, by applying MRAS to single‐cell RNA‐seq data, crucial regulatory relationships that govern cell‐type specific splicing programs have been uncovered. Overall, MRAS presented as an accurate and versatile approach to unraveling the underlying mechanism of splicing regulation.

## Graphical abstract

This study developed a computational approach called MRAS, designed to identify master regulators of splicing differences across various biological contexts. In this framework, altered RBP expression, rather than spliceosomal mutations or other modifications, plays a central role in driving these splicing differences. MRAS presented as an accurate and versatile approach to unraveling the underlying mechanism of splicing regulation.

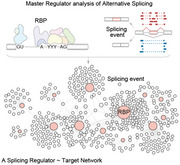

## 1 Introduction

Alternative splicing (AS), which performs on over 95% of human genes, is a crucial post‐transcriptional process that illuminates the complexity of the human transcriptomes^[^ ^1^, ^2^, ^3^, ^4^ ^]^ and contributes to functional protein diversity. Growing evidence highlights the role of aberrant splicing in various biological processes, including angiogenesis, epithelial‐to‐mesenchymal transition (EMT), and tumorigenesis.^[^ ^5^, ^6^, ^7^, ^8^, ^9^, ^10^, ^11^, ^12^ ^]^ While genetic mutations in splicing factors (SFs) have been reported as drivers of splicing dysregulations in hematologic malignancies, increasing evidence suggests that aberrant splicing can persist even in the absence of splicing factor mutations. Therefore, although mutations in splicing factors are rare in solid tumors, aberrant splicing changes are widespread and proven to promote tumorigenesis.^[^ ^13^, ^14^, ^15^ ^]^ Similarly, pediatric tumors show extensive splicing dysregulations without the presence of spliceosomal mutations.^[^ ^16^ ^]^ These observations suggest that, besides genetic mutations, other factors are likely involved in regulating cancer‐associated splicing alterations, especially in solid tumors.

In the regulation of mRNA splicing, a class of RNA‐binding proteins (RBPs) function as *trans*‐acting factors that modulate splicing by recognizing and binding to *cis*‐regulatory elements on the pre‐mRNA.^[^ ^10^, ^17^, ^18^ ^]^ The altered expression of these RBPs can also cause cancer‐associated splicing abnormalities in a concentration‐dependent manner. For instance, the serine/arginine‐rich protein SRSF1 is upregulated in many cancers and proven to promote tumorigenesis.^[^ ^17^, ^19^ ^]^ Similarly, the over‐expression of specific heterogeneous nuclear ribonucleoproteins (hnRNPs) results in the exclusion of exon 9 of pyruvate kinase muscle (PKM), leading to a switch of cellular metabolism in glioma.^[^ ^20^ ^]^ These findings suggest that the altered RBP expressions function as a potential contributor to splicing disorders. However, currently, there is no computational method available to efficiently and systematically identify key RBPs responsible for inducing context‐dependent splicing changes and driving cellular phenotypes. One naïve solution is to perform a quick differential gene expression analysis of RBPs, yet this method may not establish a direct link between the top differentially expressed RBPs and the specific splicing aberration under the biological scenario. Moreover, the traditional experimental screening approaches are certainly not feasible due to the tremendous number of splicing defects, the hundreds of RBPs involved, and the intricate interplays between them.

Our solution of inferring the top splicing regulators is inspired by the idea of master regulator analysis, a topic that has gained significant attention in recent decades.^[^ ^21^, ^22^, ^23^ ^]^ Master regulators, which contain regulatory proteins such as primarily transcription factors (TF) and co‐transcription factors, occupy the very top regulatory hierarchy within the constructed TF‐target network and collectively orchestrate the regulatory programs of the underlying cellular phenotypes.^[^ ^24^ ^]^ In fact, mRNA splicing is also orchestrated by numerous RBPs with extensive and dynamic interactions, and perturbation of one single RBP usually triggers simultaneous changes in multiple splicing events whose pre‐mRNAs share similar sequencing features or binding motifs. These complex interplays between and within RBPs and splicing events naturally form an interaction network. Thus, our aim of finding the master splicing regulators from this RBP‐events network is similar but in parallel to the idea of the well‐established master regulators analysis performed on a TF‐target network. However, we could not directly apply current algorithms to find master regulators in our splicing scenario due to incompatible inputs and biological facts. For instance, the identification of alternative splicing events requires specific software and data preprocessing, and the Percent‐Spliced‐In (PSI) values used for splicing quantification are quite different from gene expression values in terms of distribution and range.

To address the above challenges, we have developed a novel mathematical approach called MRAS (Master Regulator analysis of Alternative Splicing) to identify the critical splicing factors whose altered expression drives extensive splicing abnormalities, particularly in the biological scenario where spliceosomal mutations or other known causes of splicing disorders are absent. We validated the power of MRAS by applying it to the RNA‐seq data from the ENCODE 3 project,^[^ ^25^ ^]^ a series of cancer phenotypes as well as single‐cell RNA‐seq data. Overall, MRAS offers a robust solution to the challenge of inferring the top regulators of splicing changes in a context‐dependent manner, providing valuable insights into the mechanisms of splicing regulation.

## 2 Results

### 2.1 Network‐Based Inference of Master Splicing Regulator

**Figure 1:**
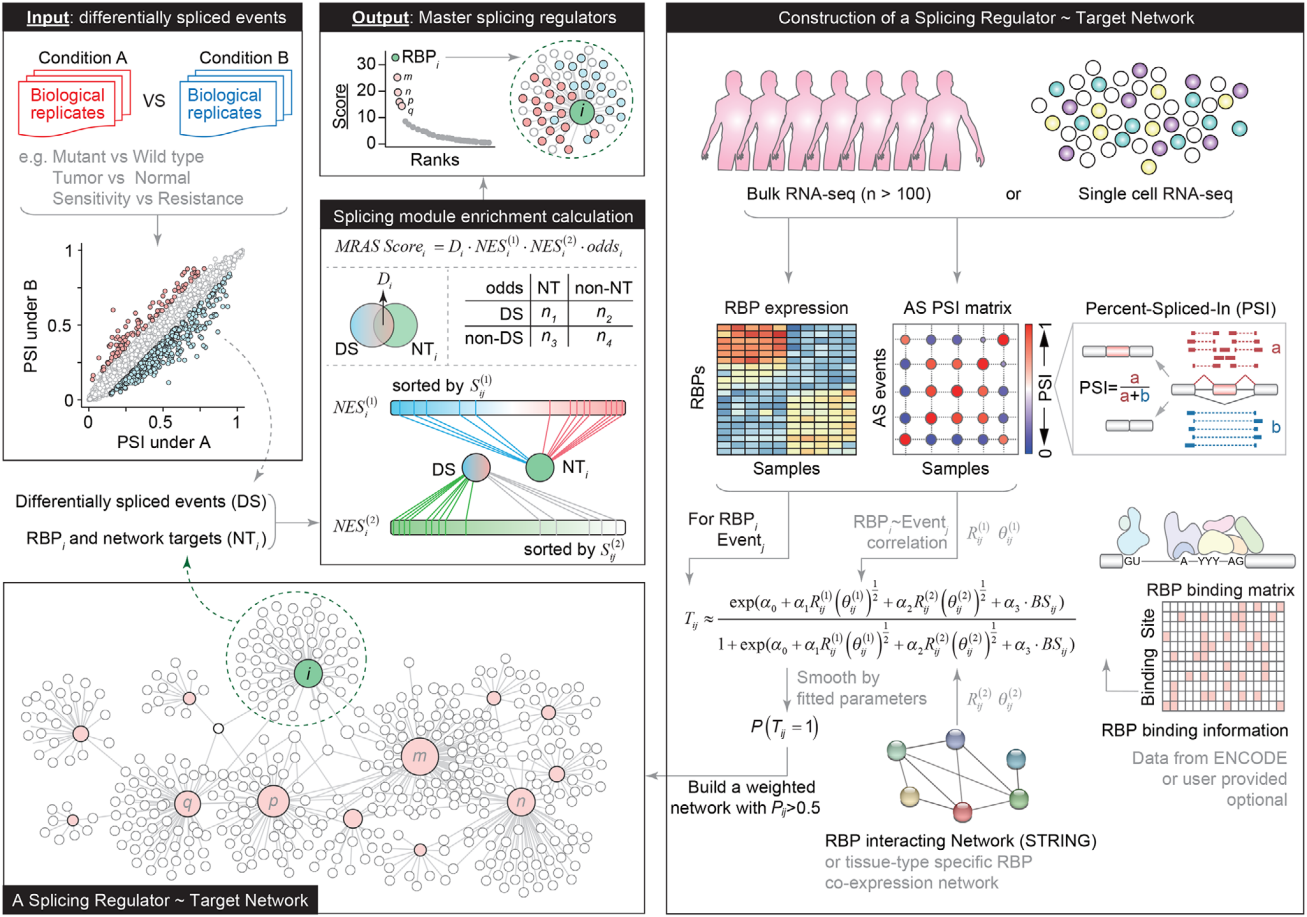
Schematic overview of the MRAS algorithm for identifying master splicing regulators. MRAS is designed to identify key RBPs that drive splicing variations and potentially influence phenotypic variability. To achieve this, MRAS pre‐constructs a cell/tissue‐specific RBP‐event regulatory networks using independent large‐scale cohorts (*n* > 100) of patient RNA‐seq data or single‐cell RNA‐seq data (Figure 1, upper right panel). This network is generated by integrating RBP expression, alternative splicing profiles, and optionally, RBP binding information (Figure 1, middle right panel). MRAS quantifies the regulatory strength of each RBP and RBP groups on splicing events using a logistic regression model, incorporating RBP‐event correlations, the STRING‐based PPI network (or optional a tissue‐type specific co‐expression network), and the RBP binding matrix (Figure 1, lower right panel). The STRING PPI network (or optional a tissue‐type specific co‐expression network) is used to estimate the co‐regulation effect among RBPs by adjusting the correlation between RBP *i* and event *j*. Finally, the relationships between RBPs and their targets are represented as the edges in the final network (Figure 1, lower left panel). In this RBP‐event regulatory network, pink and green nodes indicate RBPs, white nodes represent splicing events, and edges denote the predicted regulatory relationship. MRAS is user‐friendly and applicable across different conditions where differential splicing (DS) occurs, such as mutant versus wild type, tumor versus normal, or sensitivity versus resistance (Figure 1, upper left panel). Using the tissue type matched splicing regulatory network and DS list, MRAS calculates different enrichment scores for each RBP in the network to test that whether the DS events are significantly enriched to be the splicing regulatory targets of specific RBPs (Figure 1, middle panel, see Experimental Section). For example, as indicated by the green outline, RBP *i* and its targets (*NT_i_*) are analyzed for enrichment scores along with DS. This process is repeated for all RBPs. After systematically evaluating each RBP, MRAS generates a ranked list that highlights the most influential splicing regulators as master splicing regulator (Figure 1, upper middle panel).

To identify the master splicing factors at the top of the regulatory hierarchy, we developed MRAS, a tool designed to perform network‐based inference of key RBPs whose activity changes can lead to extensive splicing disorders and potentially influence phenotypic variability (**Figure** **1**, see Experimental Section). Briefly, MRAS pre‐constructs the cell/tissue‐specific RBP‐event regulatory networks using large‐scale cohorts of patient RNA‐seq data. RBP expressions, alternative splicing profiles, and RBP binding information are the key features to generate these networks, which serve as comprehensive references encompassing all potential splicing relationships within the background cell types. Then, based on user‐provided context‐specific splicing changes, MRAS maps the AS events onto the network and performs network‐based enrichment analysis to identify the master splicing regulators relevant to the user's biological conditions.

To construct the splicing regulatory network, MRAS begins by linking the splicing events with RBPs through correlation calculations (see Experimental Section). It then smooths and quantifies the regulatory strength of RBP on each event using a logistic regression model. To account for the effects of RBP co‐regulation, we also incorporate combinations of interacting RBPs as additional variables into the regression, which is optional for users. In the current version, MRAS provides 33 pre‐constructed networks as a resource for users developed from The Cancer Genome Atlas Program (TCGA) database (https://ngdc.cncb.ac.cn/ascancer/download/mras). These networks would better help the users to quickly perform MRAS analysis by selecting the network under the right cell type according to their own needs (Figure S1, Supporting Information). Interestingly, compared to solid tumors, acute myeloid leukemia (AML) has presented a higher percentage of cancer‐specific splicing events, which is possibly due to its unique hematologic features. Next, to identify the master regulators, MRAS performs a network‐based enrichment analysis using a list of differentially spliced (DS) events that are specific to the user's biological context. MRAS defines and integrates four scores to measure the enrichment level (see Experimental Section). The first score assesses the regulatory potential of each RBP on DS, considering three factors: the RBP expression fold change, the regulatory strength of the RBP, and the PSI changes in the splicing targets under the user's conditions (see Experimental Section). The second score is the enrichment score from a pre‐ranked GSEA analysis, in which DS events are tested against a pre‐ranked list of the RBP targets by the network weights.^[^ ^26^ ^]^ Similarly, the third score assesses the enrichment score of the RBP network targets against a pre‐ranked list of the DS events based on PSI changes between the user's two conditions. The last score is a normalized odds ratio to measure the consistency between DS and the network targets of the RBP. Finally, MRAS integrates these four scores and outputs a ranking list of top RBPs, highlighting potential master splicing regulators.

### 2.2 MARS Identifies Predefined Splicing Regulators in Simulations

**Figure 2:**
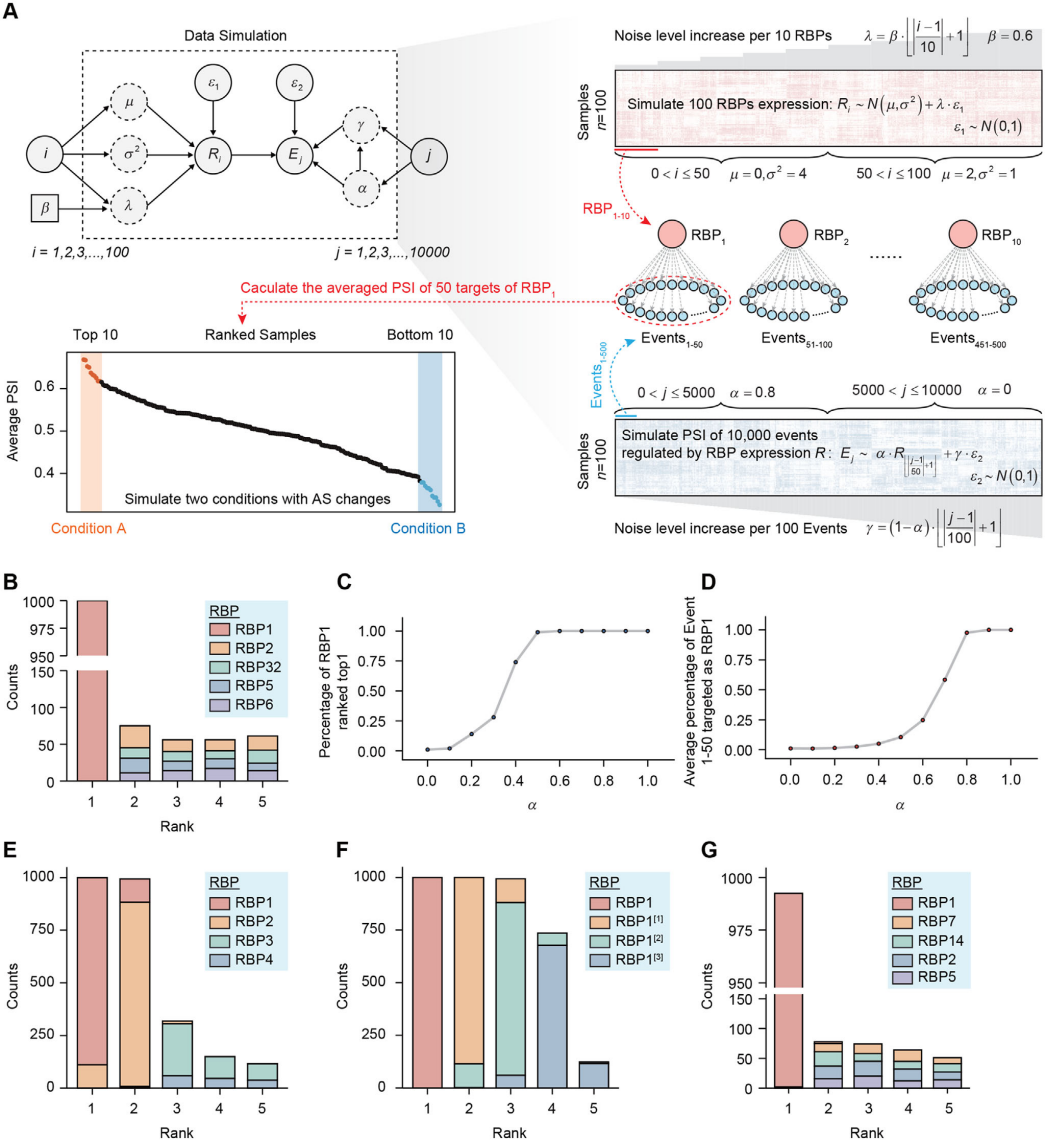
MARS identifies predefined splicing regulators in simulations. A) Overview of the simulation design, see the Experimental Section. B) Statistics of the top 5 RBPs predicted by MRAS in 1000 repeats. Only the top 5 most frequently identified RBPs were shown. C) Percentage of RBP1 identified as top one regulator by MRAS in 100 repeats at each α value. D) Average percentage of events 1–50 inferred as RBP1 targets by MRAS in 100 repeats at each α value. E) Simulation of RBPs co‐regulations by merging network targets of multiple RBPs to model the context‐specific splicing disorders. Selected network targets were events 1–40 (40 RBP1 targets), 51–80 (30 RBP2 targets), 101–120 (20 RBP3 targets), and 151–160 (10 RBP4 targets). Other settings are the same as (B). F) Simulation of additional RBPs that co‐regulate the same network targets of RBP1, see Experimental Section. RBP1,^[^ ^1^ ^]^ RBP1^[^ ^2^ ^]^ and RBP1^[^ ^3^ ^]^ are the simulated 3 additional co‐regulators of RBP1 targets. Other settings are the same as (B). G) Simulation of RBP expressions following gamma distribution. Other settings are the same as (B).

We next sought to test MRAS with simulation data, where RBPs from a constructed splicing regulatory network were pre‐selected to induce the condition‐specific splicing disorders (**Figure** **2**A, see Experimental Section). First, we designed a splicing regulatory network consisting of 100 RBPs, each regulating 50 events. Based on this framework, we simulated an RBP expression matrix and a PSI matrix for the splicing targets, in which the regulatory relationships were embedded by enforcing numerical correlations. Next, we picked up RBP1 as the ground truth of the master regulator and generated two groups of samples based on the splicing changes of the 50 network targets of RBP1. Finally, using the above inputs, we tested MRAS to determine if it could correctly identify the predefined RBP1 as the master splicing regulator. In the first simulation setting, each RBP was set to regulate 50 events, and the RBP expressions were generated under independent distributions. As a result, each RBP showed strong correlations exclusively to its own targets (Figure S2A, Supporting Information). There was no correlation between the expressions of different RBPs or between the splicing targets of different RBPs, but strong correlations were observed among splicing targets regulated by the same RBP (Figure S2B,C, Supporting Information). These results verified that our artificial matrices effectively captured the predefined regulatory associations. Next, we applied MRAS to work on these matrices for network construction and calculated the list of differentially spliced events between the two generated groups for network‐based enrichment analysis (see Experimental Section). As expected, MRAS consistently and accurately identified RBP1 as the top master splicing regulator in 1000 repeated simulations (Figure 2B).

In addition, to evaluate the sensitivity and robustness of MRAS, we repeated the calculation with varying parameters, including the mean value μ and variance σ^2^ of RBP expressions, and α_,_ which controls the strength of RBP modulations on targets. While changes in μ and σ^2^ have limited effects, we found that the performance of MRAS is heavily affected by α (Figure 2C,D; Figure S2D,E, Supporting Information). As the intensity of RBP regulation increased, MRAS demonstrated improved performance both in ranking RBP1 and in recognizing the percentage of 50 predefined events as RBP1 network targets (Figure 2C,D). The above simulations imply that MRAS can effectively identify the single key RBP based on the predefined RBP regulations. Next, to model the co‐regulations of multi‐RBPs, we modified the simulation procedures in two different settings. First, we simulated the context‐specific splicing disorders by merging network targets of multiple RBPs rather than relying solely on RBP1. Second, we introduced additional RBPs that co‐regulate the same network targets (see Experimental Section). Remarkably, MRAS could still successfully capture the ground truth as well as the predefined RBP co‐regulations in these simulations (Figure 2E,F). Interestingly, besides RBP1, the ranking and frequency of other regulatory RBPs recognized by MRAS are highly consistent with their designed contributions to the background truth (Figure 2E,F). Moreover, we have also tested MRAS under different numeric distributions of RBP expression and found that its performance remained consistent (Figure 2G). Finally, we tested whether the size of the predefined RBP targets influences the performance of MRAS (see Experimental Section). To test this, we gradually increased the number of predefined targets for RBP1 from 5 to 100. We found that MRAS struggled to identify RBP1 as the top regulator with a limited number of targets. However, as the size of predefined RBP targets increased, MRAS faithfully recapitulated RBP1 in all 100 repeats after setting network targets to 40 (Figure S2F, Supporting Information). Overall, the above results indicate the capacity and robustness of MRAS in identifying the top splicing regulators under various simulations of splicing disorders.

### 2.3 MRAS Accurately Identifies the Target RBPs from ENCODE Knockdown Experiments

**Figure 3:**
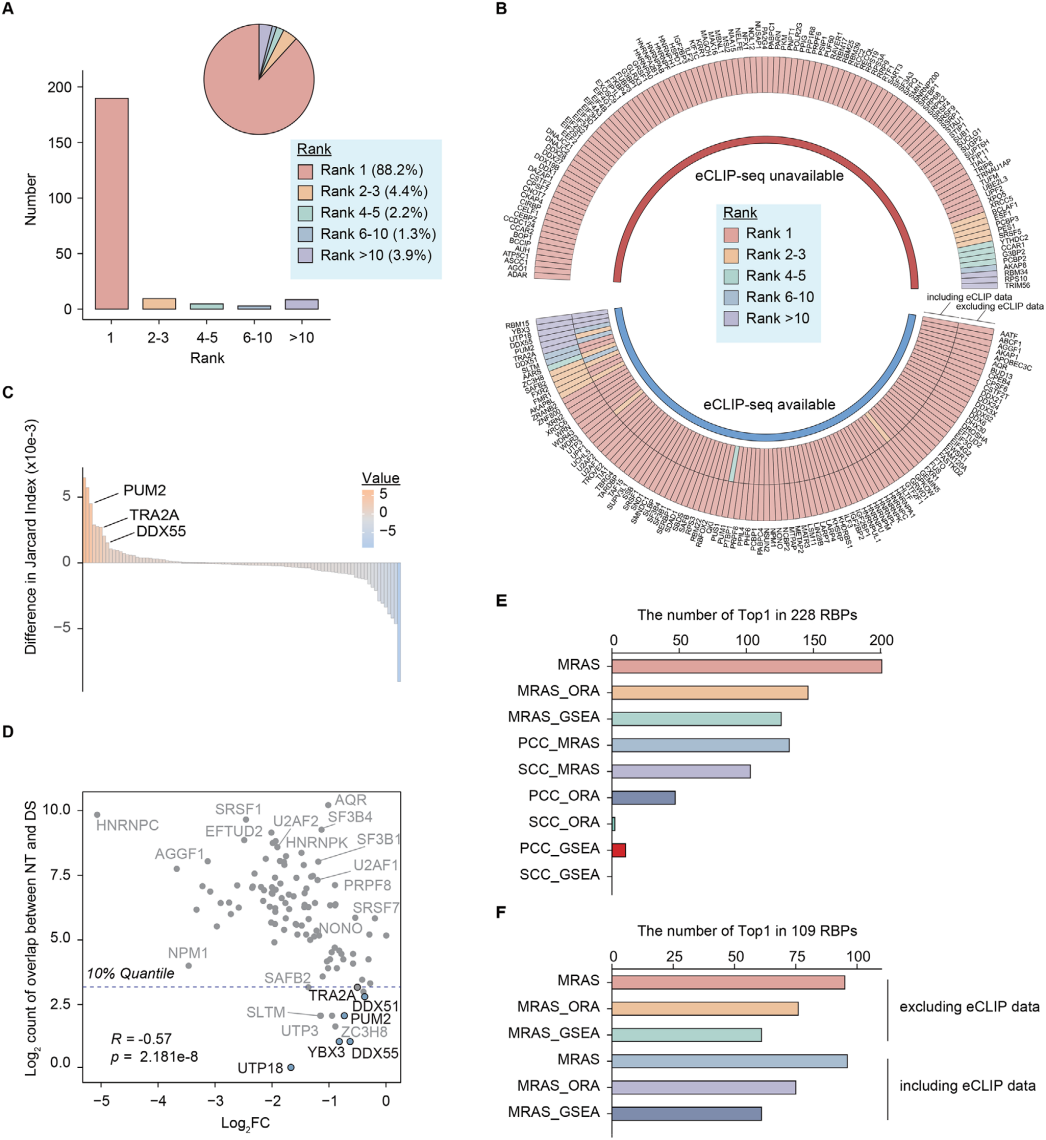
MRAS accurately identifies the target RBPs from ENCODE knockdown experiments. A) Counts of target RBPs that were successfully identified as top regulators by MRAS. Pieplot shows the distribution of different ranks. B) Circular heatmap showed the different results of MRAS by including (inner) or excluding (outer) eCLIP‐seq data. C) The changes of the Jaccard Index for each RBP by including or excluding eCLIP‐seq data. The Jaccard Index was calculated based on MRAS network targets of each RBP and differentially spliced events upon RBP knockdown. D) Scatterplots of 109 RBPs showed the log2 count of overlapped events of MRAS network targets (NT) that also get differentially spliced (DS) events upon RBP knockdown (x‐axis) and RBP expression log2 fold‐change (y‐axis). E) Ablation studies of MRAS by replacing network construction and enrichment analysis with alternative methods. F) Ablation study of MRAS for the part of enrichment analysis by including or excluding eCLIP data for the network construction.

We further tested MRAS using real RNA sequencing data from the RBP knockdown experiments on 235 RBPs as a part of the ENCODE project phase III dataset.^[^ ^25^ ^]^ The splicing changes upon RBP knockdown were used as input to test whether MRAS could correctly identify the target RBP as a key splicing regulator (see Experimental Section). We first examined the knockdown effect of each RBP by measuring the expression fold change, excluding 7 RBPs from the analysis due to very limited effect (Figure S3A, Supporting Information). We next performed differential splicing analysis by comparing knockdown cases of each RBP with control counterparts, and the results showed a diverse influence on splicing. Notably, AQR knockdown resulted in the largest number of detected AS events, which is consistent with previous report^[^ ^27^, ^28^ ^]^ (Figure S3B, Supporting Information). By screening the 228 RBP knockdown RNA‐seq, MRAS has accurately identified 88.2% (*n* = 201) of the RBPs as the top regulator, and placed 96% (*n* = 219) of RBPs within the top 10 predilections (**Figure** **3**A). Next, we tested the performance of MRAS with the integration of RBP binding information from eCLIP data for the same RBP.^[^ ^25^ ^]^ Overall, the incorporation of eCLIP data has achieved comparable performance to using RNA‐seq data alone on this ENCODE dataset (Figure 3B; Figure S3C,D, Supporting Information), which suggests that the regulatory network constructed by RNA‐seq alone is sufficient to recognize the target knocked‐down RBPs. Interestingly, 3/6 (*DDX55*, *PUM2*, and *TRA2A*) of the RBPs that failed to rank among the top 10 in previous predictions were correctly identified as top 10 regulators when integrating eCLIP data (Figure 3B). To examine the impact of including eCLIP data on the constructed regulatory networks, we calculated the overlap changes between the network targets of each RBP and differentially spliced events upon RBP knockdown. Most RBPs showed very limited changes, with *DDX55*, *PUM2*, and *TRA2A* among the top RBPs showing increased overlap after integrating the RBP binding information (Figure 3B,C). We next further examined this overlap across all RBPs and found a significant positive correlation with the RBP expression fold‐change (Figure 3D). Especially, RBPs that failed to be recognized by MRAS as the top 10 regulators had very limited overlap (Figure 3B,D). We further looked at the annotated function of all RBPs based on classifications from previous study^[^ ^25^ ^]^ and found diverse functions associated with the RBP process (Figure S4, Supporting Information). In general, RBPs that are not identified by MRAS tend to be associated with other RNA functions, as annotated by previous study.^[^ ^25^ ^]^ For example, *YBX3* (involved in RNA stability^[^ ^29^ ^]^), *UTP18* (involved in rRNA processing^[^ ^30^ ^]^), and *RBM15* (involved in RNA export^[^ ^31^ ^]^) are not identified as the top 10 regulators (Figure 3D; Figure S4, and see Experimental Section).

In addition, we performed ablation studies using this ENCODE dataset to determine the relative contribution of different parts of MRAS, namely network construction and enrichment scoring (see Experimental Section). By replacing these two parts with alternative approaches, we observed that the original procedure of MRAS achieved the best performances in accurately detecting target RBPs (Figure 3E). Incorporation of either the network construction or the enrichment scoring elements alone outperformed the models where both parts were replaced. Moreover, we tested the dependence of eCLIP data on the performance of different models. Again, on this specific ENCODE dataset, we found comparable performances by including or excluding the eCLIP data as input, and the original design of MRAS achieved the best performance than others (Figure 3F). These observations highlight the superior performance of MRAS over other strategies.

### 2.4 MRAS Identifies Key Splicing Regulators Associated with Various Tumor Phenotypes

**Figure 4:**
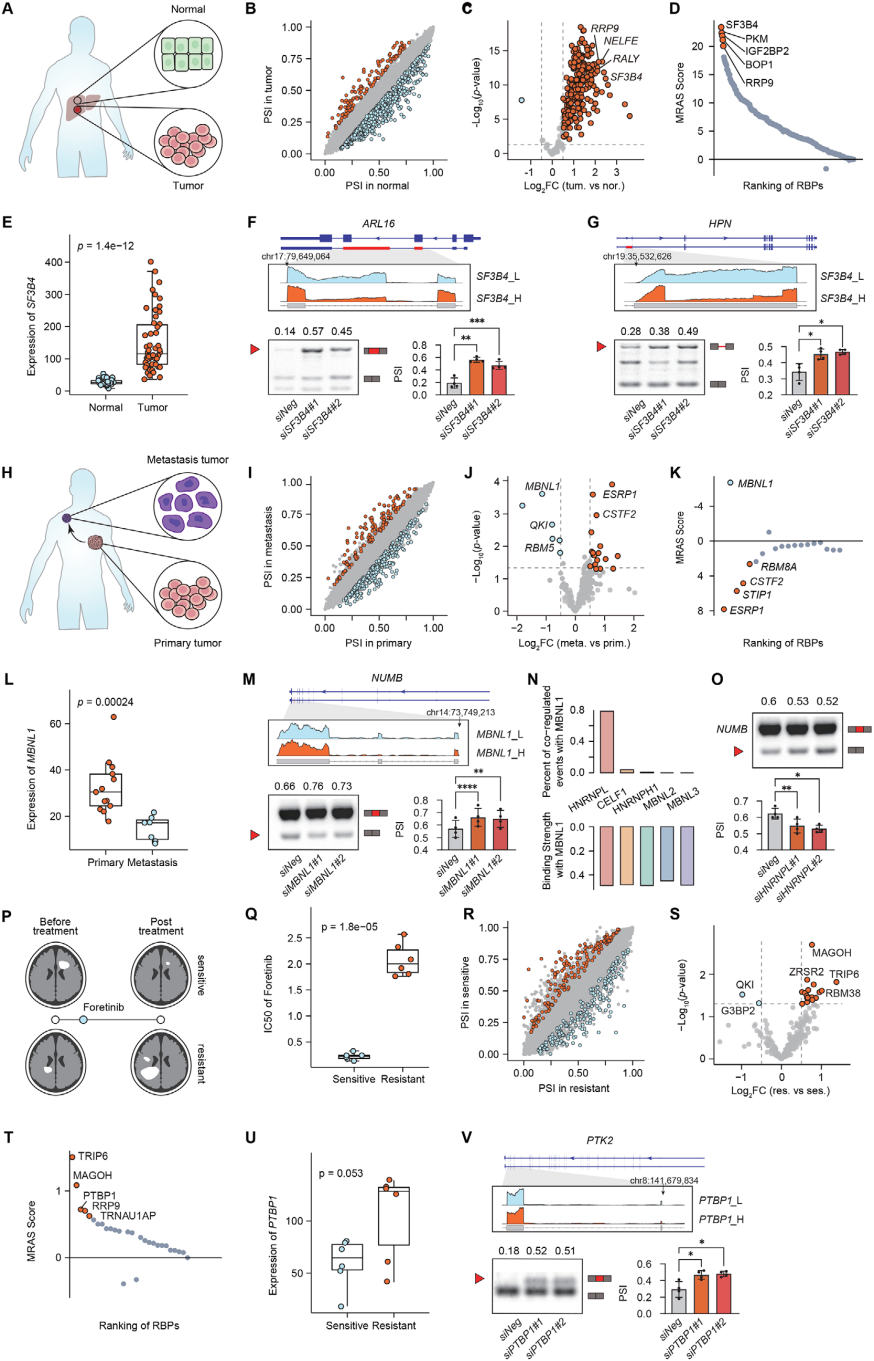
MRAS identifies key splicing regulators associated with various tumor phenotypes. A) Schema of MRAS application on comparing 50 paired normal versus tumor HCC samples. B, C) Differentially spliced events and differentially expressed RBPs between normal and tumor HCC samples. D) A ranking of splicing regulators by MRAS predictions with the top five colored red. E) Increased expression of *SF3B4* in HCC tumors. F) RT‐PCR was performed to validate AS changes of *ARL16* by *SF3B4* knockdown in Huh7 cells. G) RT‐PCR was performed to validate AS changes of *HPN* by *SF3B4* knockdown in Huh7 cells. H) Schema of MRAS application on comparing 13 primary and 7 metastasis breast cancer samples. I,J) Differentially spliced events and differentially expressed RBPs between primary and metastasis samples. K) A ranking of splicing regulators by MRAS predictions with the top five colored red or blue, which distinguish the direction of altered expression. L) Increased expression of *MBNL1* in metastasis breast cancers. M) RT‐PCR was performed to validate AS changes of *NUMB* by *MBNL1* knockdown in T47D cells. N) (up) The percentage of AS events co‐regulated with *MBNL1*. (down) The interaction strength with *MBNL1* from the STRING database. O) RT‐PCR was performed to validate AS changes of *NUMB* by *HNPNPL* knockdown in T47D cells. P) Schema of MRAS application on comparing six drug‐sensitive and six drug‐resistant glioblastoma samples treated with Foretinib. Q) IC50 value of Foretinib treatment between resistant and sensitive samples. R,S) Differentially spliced events and differentially expressed RBPs between resistant and sensitive samples. T) A ranking of splicing regulators by MRAS predictions with the top five colored red. U) Increased expression of *PTBP1* in Foretinib‐resistant tumors. V) RT‐PCR was performed to validate AS changes of *PTK2* by *PTBP1* knockdown in U87MG cells. F,G,M,O,V) PSI was quantified and the bar plot showed repeated experiment (*n* = 4).

Accumulated evidence shows that splicing aberrations contribute to the cancer process.^[^ ^1^, ^2^, ^3^, ^4^ ^]^ We applied MRAS to investigate the involvement of splicing in various stages of cancer development, including tumor initiation, metastasis, and drug resistance. Hepatocellular carcinoma (HCC), one of the most prevalent and aggressive cancers, is marked by notable splicing disruptions, which are a characteristic feature of liver disease.^[^ ^32^, ^33^ ^]^ To investigate the role of splicing in HCC neoplasia, we identified the tumor‐specific splicing alternations by comparing 50 paired normal‐tumor HCC samples, subsequently applying MRAS to identify key regulators that govern the splicing disorders during cell transformation^[^ ^34^ ^]^ (**Figure** **4**A). By this comparison, we observed extensive splicing changes in HCC cells, and more interestingly, a great number of RBPs show increased expressions in tumor cells than normal counterparts (Figure 4B,C). To ascertain the master splicing regulator, MRAS constructed the splicing regulatory network and provided a ranking score for RBPs based on the enrichment of 883 differentially spliced events. MRAS pinpointed *SF3B4* as the top regulator, which exhibited significantly upregulated expression in HCC tissue (Figure 4D,E and Table S1). However, *SF3B4* was not among the top RBPs that were mostly upregulated in HCC (Figure 4C), which implies that such splicing regulations might be overlooked in a quick differential gene expression analysis of RBPs. *SF3B4* has been reported as a poor prognosis marker^[^ ^35^, ^36^ ^]^ in various cancers. By checking the MRAS network targets of *SF3B4*, we selected some top regulated targets and further verified them experimentally. We used siRNA to knockdown *SF3B4* in the human liver Huh7 cells and found consistent splicing changes in target genes *ARL16*, *HPN*, and *DVL3* (Figure 4F,G; Figure S5A and Table S2, Supporting Information). Specifically, we observed decreased usage of short isoform of *ARL16* by silencing *SF3B4*. *ARL16, a* GTPase of the ARF family, has been implicated in different stages of tumorigenesis through alternative splicing.^[^ ^37^ ^]^

Besides tumor initiation, tumor metastasis poses a major challenge in breast cancer treatment.^[^ ^38^, ^39^ ^]^ Therefore, we compared the splicing changes and altered RBP expressions between primary (*n* = 13) and distant metastasis (*n* = 7) breast cancer tumors^[^ ^40^ ^]^ (Figure 4H,J). We adopted the network based on the TCGA BRCA RNA‐seq dataset (*n* = 1150) to test the enrichment of a list of 457 differentially spliced events between primary and distant metastasis tumors. MRAS predicted that *ESRP1 and MBNL1* are the top regulators (Figure 4K and Table S3, Supporting Information). Interestingly, we found that *MBNL1* (*n* = 104), with decreased expression in the metastasis group (Figure 4L), regulated even nearly two times different splicing events (the overlap of DS and NT) than *ESRP1* (*n* = 59), drawing significant attention. Among the network targets of *MBNL1*, we found a close association between *MBNL1* loss and the inclusion of exon 12 in *NUMB* and exon 11 in *EXOC1*. We used siRNA to knockdown *MBNL1* in human T47D cells and confirmed these regulation relationships (Figure 4M; Figure S5B and Table S2, Supporting Information). Alternative splicing of *NUMB* has been associated with proliferation, migration, and invasion in Hela and HCC cells.^[^ ^41^, ^42^ ^]^ Furthermore, when considering RBP co‐regulations, MRAS inferred that *HNRNPL* and *MBNL1* co‐regulated a substantial number of the same splicing events (Figure 4N). Interestingly, *HNRNPL* loss induces the exclusion of exon 12 in *NUMB* and the inclusion of exon 11 in *EXOC1* (Figure 4O; Figure S5C, Supporting Information), suggesting a cooperative role of these two proteins in splicing regulation during breast cancer metastasis.

Resistance to therapy is a big challenge in cancer treatment, with multifaceted^[^ ^43^ ^]^ causes. Currently, accumulated evidence shows that splicing aberrations could also contribute to this process.^[^ ^44^, ^45^ ^]^ To explore how RBP dysregulation is involved in drug resistance, specifically in glioblastoma (GBM), we collected RNA‐seq of 45 patient‐derived cells with treatment response to Foretinib, a multi‐kinase inhibitor of MET and VEGFRs^[^ ^46^ ^]^ (Figure 4P). We generated two sample sets as the sensitive and resistance groups based on the top/bottom 15% of IC50 value (Figure 4Q) and have found significant splicing changes and altered RBP expressions between them (Figure 4R,S). Next, we used MRAS to build the RBP‐events regulatory network from the TCGA GBM RNA‐seq cohort (*n* = 171) and identified *PTBP1* as one of the master splicing regulators in the resistant group (Figure 4T and Table S4, Supporting Information), with elevated expression (Figure 4U). We next explored the splicing targets of PTBP1 and found the skipping of exon 31 in *PTK2* (as known as FAK). We used siRNA to knockdown *PTBP1* in human U87MG cells and confirmed the increased exon 31 inclusion in *PTK2* (Figure 4V and Table S2, Supporting Information). As an important member of VEGF signaling, previous studies have indicated that conditional knockout of FAK can prevent VEGF‐associated angiogenesis.^[^ ^47^, ^48^ ^]^ This led us to question whether splicing alterations in FAK have an effect on angiogenesis. To test this, we performed a GSEA analysis between patients with low/high 25% of PSI changes of *PTK2* cassette exon 31 from the TCGA GBM cohort, and found a significant enrichment of angiogenesis pathway in patients with *PTK2* exon 31 skipping, which is more presented in our Foretinib resistant group (Figure S5D,E, Supporting Information). Based on these observations, we suspect that when VEGF‐associated angiogenesis is inhibited by Foretinib, tumor cells will seek to activate angiogenesis by introducing this splicing variant of *PTK2*. However, this is only our hypothesis of how splicing changes regulated by *PTBP1* may link to Foretinib resistance, which required independent validation in further experimental study.

In addition to above identifed novel splicing regulatory relationships, we also evaluated the effectiveness of MRAS to additional disease contexts where key splicing factors are well‐known to drive the disease phenotypes (Figures S6 and S7, Supporting Information). Specifically, we applied MRAS to glioblastoma (GBM, EGAS00001002515) and Alzheimer's disease (AD, GSE173955) to assess its ability to identify well‐known splicing regulators. In GBM, MRAS successfully identified *PTBP1*, a key splicing regulator known to influence the mutual exclusive selection of exon 9 and exon 10 of PKM, which is highly consistent with published studies^[^ ^20^, ^49^, ^50^ ^]^ (Figure S6, Supporting Information). Similarly, in AD, MRAS identified *ELAVL2* and *ELAVL4* as the top splicing regulators which are well‐established splicing regulators in AD^[^ ^51^, ^52^, ^53^ ^]^ (Figure S7, Supporting Information).

### 2.5 Application of MRAS on Single Cell RNA‐Seq Datasets

Bulk RNA sequencing of heterogeneous tissues represents a mixed signal from different cell types, so transcriptome profiling on single‐cell resolution becomes the common approach to dissect this heterogeneity.^[^ ^54^ ^]^ Within individual cell clusters, cell type‐specific alternative splicing signatures frequently emerge,^[^ ^55^ ^]^ suggesting that single‐cell AS profiles may reveal intrinsic RNA splicing heterogeneity. Here, we demonstrated the application of MRAS on single‐cell RNA seq datasets.

**Figure 5:**
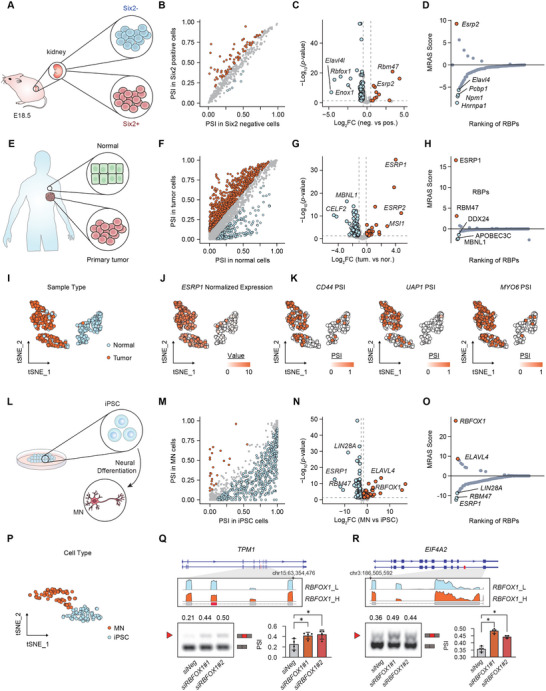
Application of MRAS on single‐cell RNA‐seq datasets. A) Schema of MRAS application on single‐cell RNA‐seq data by comparing Six2 negative (*n* = 288) and positive (*n* = 288) kidney cells in mice. B,C) Differentially spliced events and differentially expressed RBPs between Six2 negative and positive kidney cells. D) A ranking of splicing regulators by MRAS predictions with the top five colored red or blue, which distinguish the direction of altered expression. E) Schema of MRAS application on single‐cell RNA‐seq data by comparing tumor (*n* = 326) and normal (*n* = 201) breast cancer cells. F,G) Differentially spliced events and differentially expressed RBPs between tumor and normal cells. H) A ranking of splicing regulators by MRAS predictions with the top five colored red or blue, which distinguish the direction of altered expression. I) t‐SNE plot based on PSI values of *ESRP1* targets. J) t‐SNE plot with cells colored by the expression of *ESRP1*. K) t‐SNE plot with cells colored by PSI of *CD44*, *UAP1*, and *MYO6*, the targets of *ESRP1*. L) Schema of MRAS application on iPSC (*n* = 63) and MN (*n* = 70) cells. M, N) Differentially spliced events and differentially expressed RBPs between iPSC and MN cells. O) A ranking of splicing regulators by MRAS predictions with the top five colored red or blue. The MRAS score of RBP with increased expression was positive, and vice versa. P) t‐SNE plot based on PSI values of *RBFOX1* targets. Q) RT‐PCR was performed to validate AS changes of *TPM1* by *RBFOX1* knockdown in U87MG cells. R) RT‐PCR was performed to validate AS changes of *EIF4A2* by *RBFOX1* knockdown in U87MG cells. Q), and R) PSI was quantified and the bar plot showed repeated experiment (*n* = 4).

Kidney development is a complex process involving multiple interacting cell types.^[^ ^56^ ^]^ During this process, progenitor cells undergo a mesenchymal‐to‐epithelial transition (MET) and ultimately differentiate into different tubular segments of the nephron.^[^ ^56^ ^]^ The expression of *Six2* is maintained throughout mouse kidney development, and the changes in *Six2* expression are accompanied by the transformation of cell types.^[^ ^56^, ^57^ ^]^ Consistent with previous studies,^[^ ^56^ ^]^ we observed splicing changes together with altered RBP expressions by comparing *Six2*+ and *Six2*‐ cell subgroups (**Figure** **5**A–C). Interestingly, MRAS highlighted *Esrp2* as the master regulator under kidney cell development (Figure 5D; Figure S5F and Table S5, Supporting Information), aligning with the recent finding that *Esrp2* regulates isoform switching of epithelial‐associated proteins in kidney development.^[^ ^58^ ^]^ The second application is to investigate the splicing changes in breast cancer cell transformation by single‐cell RNA‐seq data from breast cancer patients.^[^ ^59^ ^]^ Here, we observed numerous abnormal splicing events and significant changes in RBP expression at the single‐cell level of breast tumors. (Figure 5E–G). By the cell‐specific splicing network and 1037 AS events, MRAS consistently highlighted *ESRP1* as the top splicing regulator with upregulated expression in tumor cells (Figure 5H; Figure S5G and Table S6, Supporting Information). Based on the PSI values of *ESRP1* targets inferred by MRAS, tumor cells could be clearly distinguished from normal cells (Figure 5I). Among the network targets of *ESRP1*, we found several well‐studied splicing targets, including *CD44* ^[^ ^60^ ^]^, *UAP1*.^[^ ^61^ ^]^ and *MYO6*,^[^ ^61^ ^]^ which exhibited exclusive presentation in tumor cells (Figure 5J,K). The result emphasized the potential engagement of splicing disorders driven by increased *ESRP1* expression in breast cancer cell transformation.^[^ ^60^, ^61^, ^62^ ^]^ Finally, we applied MRAS onto a single cell RNA seq data to investigate the splicing dysregulation during the neuron development process, which has been previously reported to be regulated by splicing in a tightly controlled manner^[^ ^63^ ^]^ (Figure 5L). By comparing induced pluripotent stem cells (iPSCs) and motor neurons (MNs), we observed extensive splicing changes, especially exon skipping events, along with multiple dysregulated RBPs (Figure 5L–N). To investigate the master splicing regulator of 586 the AS events, we constructed the regulatory network by MRAS and identified *RBFOX1* as the master regulator, with significantly increased expression in MN cells (Figure 5O; Figure S5H and Table S7, Supporting Information). In addition, we adopted the splicing changes of the network targets of *RBFOX1* to perform t‐SNE reduction, which clearly distinguishes MN cells from iPSC cells (Figure 5P; Figure S5I, Supporting Information). This analysis also demonstrates the power of utilizing splicing features in guiding single‐cell classification. We next used siRNA to knockdown *RBFOX1* and performed RT‐PCR experiments in human U87MG cells to validate MRAS inferred splicing events, confirming exclusive splicing in MN cells on targets such as *TPM1 and EIF4A2* (Figure 5Q,R; Figure S5J,K and Table S2, Supporting Information). Notably, *TPM1* has been reported to influence neuronal cell differentiation through its alternative splicing,^[^ ^65^ ^]^ and *RBFOX1* is known to exert a growth‐suppressive effect on neuronal cells by the regulation of *TPM1* splicing isoforms.^[^ ^64^, ^65^ ^]^

## 3 Discussion

There are multiple strategies to improve MRAS. First, the current algorithm does not distinguish between direct and indirect RBP regulations, the latter may present additional edges that can result in excessive network connectivity. Besides the increased computational burden, such a network may contain too many redundant regulation relationships, potentially disrupting the network topology for the identification of master splicing regulators at the top of the network hierarchy. The implementation of network pruning could help to remove the indirect and false positive edges, thereby refining the network. Several well‐established methods could be employed for this purpose, including the use of conditional mutual information, which is already utilized in master regulator analysis.^[^ ^21^, ^22^ ^]^ Second, the current version of MRAS relies on the STRING PPI network to define the candidates of the RBP co‐regulator. Alternative approaches should be considered to increase the reliability of RBP co‐regulation prediction. For instance, integrating the PPI network with a cell‐type specific MRAS network and detecting the local network community may help to find co‐regulators specific to certain splicing processes and functions. Third, the MRAS network was used as a comprehensive reference, encompassing all potential splicing relationships within the background cell types. Thus, the construction of this network requires a large number of samples (*n* ≥ 100), which should be independent of the user's input. Currently, MRAS supports 33 types of tumors with specific background networks. Due to the limited cases of public RNA‐seq data, network construction on some types of rare tumors or cell types remains challenging. Besides, a comprehensive database of these splicing networks is also required to keep pace with the development and updates of MRAS. And the investigation of these networks enables a better understanding of the cell‐type‐specific RBP regulations as well as the ubiquitous splicing regulations that are commonly presented in nearly all cell types. Lastly, In the current design of MRAS, we did not capture regulatory relationships from other omics levels, such as DNA somatic lesions, copy number variations, and methylation. We will extend our method to integrate other types of genomic data and explore the relationships between multi‐type genomic factors and splicing events in future work.

In addition to the above computational improvements, utilization of MRAS may help to provide insights into the splicing biology in cancer genomics. For instance, we could use MRAS to define a core set of RBPs whose altered expression can lead to pathogenic splicing defects that are the most commonly observed in human cancers. And any further subdivision of these RBPs based on splicing mechanisms and substrates is also worth exploring. Moreover, MRAS facilitates the discovery of the precise distribution of splicing dysregulations by altered RBP expressions as well as the specificity and similarity of these dysregulations across human pan tumors. We could also investigate the basic characteristics of these dysregulations by altered RBP expressions and make a comparison to that of recurrent spliceosomal mutations. This will help us to understand the preference between genetic mutations and expression changes of splicing factors favored by specific types of tumors.

In summary, we have developed a computational approach called MRAS to identify master regulators of splicing differences in various biological applications, where altered RBP expression, rather than spliceosomal mutations or other alterations, plays a central role in driving these differences. With the increasing evidence of cancer‐associated splicing abnormalities, we envision that our method will shed light on the mechanisms of splicing dysregulations and associated tumorigenesis in human cancers.

## 4 Experimental Section

### The MRAS Model

MRAS was designed to identify key RBPs that lead to a set of splicing variants between different cell phenotypes. The principle of MRAS was to enrich the context‐specific splicing alternations with the RBP targets derived from a global RBP‐events regulatory network. The input for MRAS consists of two key matrices: the RBP expression matrix and the Percent Spliced In (PSI) matrix. The RBP expression matrix contains the expression levels of RBPs, while the PSI matrix contains the PSI values for each splicing event across all samples. For the RBP expression values, users can utilize either TPM or log2(TPM + 1). For the PSI values, users can generate this matrix using splicing detection tools such as rMATS^[^ ^66^ ^]^ or other splicing identification software. MRAS was designed to support any type of splicing event and allows users to quickly process PSI matrices derived from the output of different software (such as rMATS, MISO). Further details on the required input formats, data processing steps, and the definitions of specific identifiers can be found on the MRAS GitHub page. The main procedures of MRAS include two steps: network construction, and enrichment scoring.

### Network Construction

According to the tumor/cell type that the user observed splicing differences, a substantial cohort (*n* ≥ 100) of patient RNA‐Seq data with the same tumor type was first collected to construct the background network. This data was used as the foundation for constructing the regulatory network. For RBP *i*, samples were sorted by their expression and took the samples of top/bottom expression groups using a user‐defined threshold (e.g., top/bottom 10%). Then, differential splicing analysis was performed between these two groups, and events with 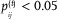 (*t*‐test) and the absolute value of dPSI (the difference of PSI between two groups) $\theta_{ij}^{\left(1\right)}>0.1$ were selected as the potential network targets of RBP *i*. It was explicitly mentioned that the differential splicing analysis in MRAS was performed using a *t*‐test to calculate the significance of splicing events between two conditions (User‐provided, such as tumor versus normal). It had been clarified that the dPSI (difference in PSI values between the two conditions) was calculated to quantify the magnitude of the splicing difference. The statistical analysis was carried out using the ‘stats’ package in R for differential splicing testing. By doing so, a prior RBP‐events regulatory network *T* had been obtained, where 
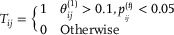
.

To further quantify the regulatory strength, a logistic regression model was used to integrate all information associated with RBP‐events regulatory activity, including the correlation, RBP‐RBP interaction, and the binding affinity between RBP and events. Specifically, $R_{ij}^{\left(1\right)}$ presents the absolute value of the Spearman correlation coefficient between RBP *i* expression and PSI of event *j*, and only significant pairs with 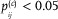 from the correlation test which was user configurable were kept.

As RBPs were involved in regulating the splicing process by interacting with each other, the same splicing event could be regulated by several RBPs simultaneously. To capture this RBP co‐regulation, an RBP‐RBP physical interaction network was extracted from the STRING protein‐protein interaction network^[^ ^67^ ^]^ (or optional a tissue‐type specific co‐expression network). For RBP *i* and event *j*, the regulatory complex was considered as all RBPs of the network neighbors of RBP *i* that also correlated with event *j*. Therefore, the complex generated by RBP *i* regulating *j* was defined as $M_{ij}=\left\{k|R_{kj}^{\left(1\right)}>R_{auto},RIN_{ki}=1\right\}\cup \left\{i\right\}$, where *RIN* was the adjacent matrix of the extracted RBP‐RBP subnetwork and *R_auto_* was determined as the upper 95th percentile (corresponding to a *p*‐value < 0.05) of the overall distribution of all RBP‐event correlations. For each pair of RBP and splicing event, the correlation was calculated between RBP expression and the PSI value of splicing event. The overall expression activity of *M_ij_* was defined as $Act_{M_{ij}}=\prod_{m}^{M_{ij}}Expr_{m}$, where *Expr_m_* was the expression level of RBP *m*. Following the same process as described above, then calculated the $R_{ij}^{\left(2\right)}$ and $\theta_{ij}^{\left(2\right)}$. $R_{ij}^{\left(2\right)}$ was the absolute value of the Spearman correlation coefficient between $Act_{M_{ij}}$ and PSI value of event *j*. $\theta_{ij}^{\left(2\right)}$ was the absolute dPSI value of event *j* under the two groups of high/low $Act_{M_{ij}}$ value.

Furthermore, the RNA binding affinity of RBPs was also contributed, which was optional for the modeling. The binding intensity matrix of RBP on splicing events was defined as *BS_ij_* ∈ *R* ^*r* × *s*^, where *r* was the number of RBPs, and *s* was the number of events. *BS_ij_* reflects the binding strength of the RBP *i* binding peak within the coordinates of event *j*, which could be estimated by the Irreproducibility Discovery Rate (IDR). The peaks by eCLIP data of 120 RBPs were directly downloaded and integrated from the ENCODE dataset (ENCSR456FVU) to generate the *BS_ij_* matrix based on the local IDR value. Users could also provide their RNA binding affinity matrix according to their cell/tumor type and sequencing method.

To integrate all the features above, a logistic regression model was trained as

(1) $$T_{ij}\approx \frac{\exp(\alpha_{0}+\alpha_{1}R_{ij}^{\left(1\right)}{\left(\theta_{ij}^{\left(1\right)}\right)}^{\frac{1}{2}}+\alpha_{2}R_{ij}^{\left(2\right)}{\left(\theta_{ij}^{\left(2\right)}\right)}^{\frac{1}{2}}+\alpha_{3}\cdot BS_{ij})}{1+\exp(\alpha_{0}+\alpha_{1}R_{ij}^{\left(1\right)}{\left(\theta_{ij}^{\left(1\right)}\right)}^{\frac{1}{2}}+\alpha_{2}R_{ij}^{\left(2\right)}{\left(\theta_{ij}^{\left(2\right)}\right)}^{\frac{1}{2}}+\alpha_{3}\cdot BS_{ij})}$$

All RBP‐events pairs were used to train the model where the RBP‐events pairs with *P*(*T_ij_* = 1) > 0.5 were positive samples, while others were negatives. The predicted *P*(*T_ij_* = 1) was used as the regulatory strength between RBP *i* and event *j*. Finally, only the edges were kept with *P*(*T_ij_* = 1) > 0.5 in the final network.

Based on the above procedure, MRAS pre‐computed with 33 cancer type‐specific splicing regulatory networks, trained by cancer patient RNA‐seq data from the TCGA datasets. Users can access these 33 networks from the AScancer Altas database (https://ngdc.cncb.ac.cn/ascancer/download/mras). MRAS also offers the option of network construction based on specific datasets provided by the users.

### Enrichment Scoring

To identify potential regulatory RBP for observed splicing events set*DS* in a case‐control study, four scores were defined based on the constructed network to assess the regulatory power of each RBP. For each RBP *i*, the first score *D_i_* presented the regulatory potential on *DS* which was consist of *P* (*T_ij_* = 1) the network regulatory weights between RBP *i* and event *j*, $\theta_{j}^{\left(0\right)}$ the absolute value of dPSI and *FC_i_* the expression fold change of RBP *i* between the user's conditions. And *D_i_* was formulated as follows:

(2) $$D_{i}=\sum_{j}\left(\theta_{j}^{\left(0\right)}\cdot P\left(T_{ij}=1\right)\right)\cdot \left|\log_{2}FC_{i}\right|$$

where *j* presents the overlap events between *DS* and network events of RBP *i*.

The second and the third scores were two enrichment scores from a pre‐ranked GSEA analysis to measure the mutual enrichment between DS and the network targets of each RBP (*NT_i_*). Specifically, two normalization terms were designed and added to the weight calculation of each splicing event. And ranked splicing events with considering these two terms. The number of regulators of each event was first normalized according to the global distribution of the network RBP‐events connections, see definition (3). This normalization emphasizes the unique regulation of the splicing events, that a more specific regulatory relationship will contribute to a larger enrichment score.

(3) $${W_{j}}^{\left(d\right)}=1+\sqrt{\frac{\max_{j}\left(d_{j}\right)-d_{j}}{\max_{j}\left(d_{j}\right)-\min_{j}\left(d_{j}\right)}}$$

where *d_j_* represents the total number of regulators of event *j* in the network. Then for each RBP *i*, the splicing changes of each target were normalized by the overall changes of all network targets *NT_i_*, see definition (4). By this second normalization, events with larger splicing differences were regarded as more reliable targets of the RBP and made a greater contribution to the enrichment score.

(4) $${W_{ij}}^{\left(\theta \right)}=1+\sqrt{\frac{\theta_{ij}-\min_{j}\left(\theta_{ij}\right)}{\max_{j}\left(\theta_{ij}\right)-\min_{j}\left(\theta_{ij}\right)}}$$

where θ_*ij*_ represents the absolute value of dPSI of event *j* between the top/bottom expression groups of RBP *i*. Next, we calculated $NES_{i}^{\left(1\right)}$, as the enrichment level of *NT_i_* in the set of *DS*, that the events of *NT_i_* were tested against a pre‐ranked list of the *DS* events sorted by a weighted score $S_{ij}^{\left(1\right)}={W_{j}}^{\left(d\right)}\cdot {W_{ij}}^{\left(\theta \right)}\cdot \theta_{j}^{\left(0\right)}$. Similarly, the enrichment level of *DS* in the *NT_i_* was calculated as $NES_{i}^{\left(2\right)}$, that the DS events were tested against a pre‐ranked list of *NT_i_* sorted by a weighted score $S_{ij}^{\left(2\right)}={W_{j}}^{\left(d\right)}\cdot {W_{ij}}^{\left(\theta \right)}\cdot P\left(T_{ij}=1\right)$. Lastly, to quantify the consistency of splicing events between *DS* and *NT_i_*, we calculated a normalized odds ratio (OR) by $odds_{i}=\sqrt{\max(x,OR_{i})}$, where *x* was defined by the user. The final enrichment score was the product of all four scores as $MRASScore_{i}=D_{i}\cdot NES_{i}^{\left(1\right)}\cdot NES_{i}^{\left(2\right)}\cdot odds_{i}$. By sorting all RBPs based on *MRASScore*, MRAS output a ranking list of top RBPs as potential splicing regulators. MRAS outputs a ranking list of top RBPs as potential splicing regulators, together with the number of network targets of each RBP.

### In Silico Simulation of Splicing Disorders by Predefined Regulators

To evaluate the performance of MRAS, a profile of condition‐specific splicing changes induced by predefined splicing regulators was simulated. In brief, a large‐scale splicing regulatory network was simulated from which each regulator was associated with a predetermined set of splicing targets. By these designs of prior associations, an RBP expression matrix was generated together with a matched PSI matrix for the splicing targets. Then, regulators were selected from these regulatory networks as default drivers and used their network targets to generate the splicing changes in a condition‐specific manner, which presents as the observation and input provided by the user. Finally, it was tested whether MRAS could faithfully recover the default drivers as master splicing regulators.

More specifically, an expression matrix of 100 RBPs on 100 samples was first generated (Figure 2a). The first 50 RBPs were simulated with variable expressions that account for the splicing diversities, while the second 50 RBPs represent housekeeping factors to maintain the splicing fidelity. Thus, for RBP *i*, its expression value *R_i_* follows normal distributions as:

(5) $$R_{i}∼N\left(\mu ,\sigma^{2}\right)+\lambda \cdot \varepsilon_{1}\;\;\;\;\;\left\{\begin{array}{c} \mu =0,\sigma^{2}=4\;\;\;0<i\leq 50\\ \mu =2,\sigma^{2}=1\;\;\;50<i\leq 100 \end{array}\right.$$

where ε_1_ was a random expression noise by ε_1_ ∼ *N*(0, 1), and λ indicates the level of noise gradients that increases per 10 RBPs.

(6) $$\lambda =\beta \cdot \left\lfloor \left|\frac{i-1}{10}\right|+1\right\rfloor$$

The PSI matrix of 10000 splicing targets on 100 samples was then simulated according to the predefined regulatory relationships with the above RBP expressions. For each of the 100 RBPs, 50 events were assigned as the true splicing targets by simulating the PSI values correlated to the RBP expressions. Therefore, for event *j*, the PSI value*E_j_* can be simulated as:

(7) $$E_{j}∼\alpha \cdot R_{\left\lfloor \left|\frac{j-1}{50}\right|+1\right\rfloor }+\gamma \cdot \varepsilon_{2}$$

Here, α represents the strength of RBP modulations, ε_2_ was the white noise given by ε_2_ ∼ *N*(0, 1), and γ controls the noise levels that increase linearly per 100 events. In so doing, 5000 events regulated by 100 RBPs were generated. Moreover, another set of 5000 events was simulated to model the background splicing noises that were not targets of any RBP by setting α equal to 0. All PSI values were lastly normalized to 0–1 as the value range of splicing variances.

(8) $$\gamma =\left(1-\alpha \right)\cdot \left\lfloor \left|\frac{j-1}{100}\right|+1\right\rfloor \;\;\;\;\;\left\{\begin{array}{c} \alpha =0.8\;\;\;\;\;0<j\leq 5000\\ \alpha =0\;\;\;\;\;5000<j\leq 10000 \end{array}\right.$$

Next, to model the splicing disorders as the interest of the user, RBP1 was pre‐set as the default master splicing regulator in the simulation study. All 100 samples were calculated and ranked by the averaged PSI of the 50 splicing targets of RBP1 and selected the top and bottom ten ranked samples to model the samples under the two different biological conditions with splicing disorders. To assess the sensitivity and robustness of MRAS, the calculation was repeated under different parameter settings 100 times each.

Moreover, to better model the complex splicing regulations in real biological scenarios, multiple RBPs, rather than only RBP1, were also pre‐set as the default master splicing regulators. To achieve this, all 100 samples were calculated and ranked by the averaged PSI of a set of selected events, consisting of events 1–40, 51–80, 101–120, and 151–160, which were regulated by RBP1, RBP2, RBP3, and RBP4 as predefined. The number of selected targets was set to decrease from RBP1 (40 targets) to RBP4 (10 targets) and then followed the same procedure as described above.

Next, the RBP co‐regulations were also modeled in another way. To this end, for each RBP *i*, three additional RBPs were added that partially copy the original expression of RBP *i* as follows:

(9) $$R_{i}^{\left[m\right]}∼\varphi_{m}\cdot R_{i}+\lambda \cdot \varepsilon_{1}\varphi_{m}=\left\{\begin{array}{c} 0.8m=1\\ 0.6m=2\\ 0.4m=3 \end{array}\right.$$

Here φ_*m*_ represents three different levels of correlations with the expression of the initial 100 RBPs. λ and ε_1_ were the same as described in definition (5). Other steps followed the same procedure as described above.

Finally, it was tested whether the size of the predefined RBP targets influences the performance of MRAS. To test this, the number of predefined targets of RBP1 was gradually increased from 5 to 100, increasing by 5 each time, and followed the same procedure described above.

### ENCODE Data Processing and Integration

shRNA/CRISPR RNA‐seq data for 235 RBPs as well as 29 normal controls of the K562 cell line were obtained from the ENCODE3 project.^[^ ^25^ ^]^ The reads mapped to each RBP were counted using FeatureCounts (v2.20)^[^ ^68^ ^]^ on the 528 BAM files and calculated TPM to obtain the RBP expression matrix. Log2 fold change was used to measure the knockdown efficiency, and 7 RBPs with log_2_FC<0.1 were filtered out with insufficient knockdown efficiency for downstream analysis. The rMATS (v4.1.2)^[^ ^66^ ^]^ was used to detect alternative splicing events and calculated PSI values to obtain the AS‐PSI matrix. To construct the RBP‐events regulatory network, samples were selected with the top/bottom 10% of expression fold change for each RBP among all 528 samples to calculate the correlation with AS events. The final regulatory network contains predicted splicing targets of 228 RBPs.

### Ablation Studies of MRAS on the ENCODE Dataset

To evaluate the contribution of the network construction and enrichment methods of MRAS, the performance of MRAS was examined by replacing these two parts with alternative strategies. For network construction methods, two alternative approaches to determine the RBP‐target edges are: 1) Pearson's correlation coefficient (PCC) > 0.5 and correlation test with *p*‐value < 0.05. 2) Spearman's correlation coefficient (SCC) > 0.5 and associated *p*‐value < 0.05. The first approach measures the linear relationship between RBP expression and PSI values, while the second approach accounts for non‐linear relationships by utilizing Spearman's rank correlation. Both approaches were used to identify significant RBP‐target interactions. For enrichment analysis, two alternative approaches were also tested: 1) ORA: The odds ratio was calculated from a Fisher Exact test to measure the over‐representation of DS in the network targets of each RBP. This method identifies the statistical significance of the enrichment of RBP‐target interactions among the splicing events that show differential splicing between conditions. 2) GSEA: This approach evaluated the enrichment of the network targets of each RBP along the pre‐ranked list of DS events ordered by dPSI value, and then used the enrichment score for master splicing regulator identification. The enrichment score (ES) derived from this analysis was used to identify master splicing regulators, with a higher ES indicating stronger enrichment of network targets among the DS events. These two complements of MRAS were replaced with different combinations of alternates, and evaluated the performance on the ENCODE RBP knockdown dataset with or without matched eCLIP data.

### RNA‐Seq Data Preprocessing

FASTQ files were downloaded from the Sequence Reads Archive (SRA) or European Genome‐phenome Archive (EGA), and aligned to the mouse (mm9) or human genome (hg19) using STAR (version 2.7.10.a).^[^ ^69^ ^]^ Raw count tables were obtained by FeatureCounts (version 2. 20),^[^ ^68^ ^]^ and the raw counts of reads for each gene were converted to transcripts per million (TPM). The RBPs used in the applications were taken from a previously published study,^[^ ^70^ ^]^ annotated as “RBP/SF.” For the ENCODE dataset, the list was supplemented with the union of the RBPs from the study and the experimental RBP knockdowns. For bulk RNA seq, alternative splicing events were detected by rMATs.^[^ ^66^ ^]^ For single‐cell RNA seq, Outrigger was used for the identification and qualification of alternative splicing events^[^ ^63^ ^]^ in the second and third applications. As for the first application, a pseudo‐bulk RNA seq was first constructed by summing the reads of all cells and used to detect the pool of alternative splicing events. Then, the splicing changes of AS events were quantified by the corresponding junction reads from each cell. For the PSI matrix, events with PSI values of NA/0/1 present on more than 80% of samples or with less than ten junction reads present on more than one‐third of the samples were filtered out in bulk RNA seq. In single‐cell RNA seq, these thresholds were relaxed.

### Cell Culture and Transfection

T47D, U87MG, and Huh7 cells were cultured in Dulbecco's modified Eagle's medium (DMEM; Gibco), and all cells were supplemented with 10% fetal bovine serum (FBS; VivaCell) and 1% penicillin–streptomycin and were incubated under 37 °C and 5% CO_2_ conditions. Cells were plated in 12‐well plates at a density of 1 × 10^5^ cells per well until 70–80% cell confluence and then were harvested to conduct transient transfection. Subsequently, the cells were transfected with either siRNA (genepharma) using Lipofectamine 2000 (Invitrogen) according to the manufacturer's protocols. The target gene expression was then analyzed in cells transfected for 24 and 36 h. The siRNA sequences are listed in Table S2 (Supporting Information).

### RNA Extraction, RT‐PCR, and qRT‐PCR

Total RNA was extracted using Trizol reagent (Invitrogen). Reverse transcription was performed in a reaction mix containing 1 µg of total RNA, using a cDNA Synthesis Kit (Lablead) according to the manufacturer's guidelines. cDNA was used as the template for both conventional PCR analysis (HotStart Taq; Novoprotein) and quantitative real‐time PCR analysis (SYBR Green method; Lablead). PCR primers are listed in Table S2 (Supporting Information).

### Statistics of PCR Results

PSI values of each event was quantified using ImageJ. Statistical comparisons for PCR results were performed using paired Student's *t*‐test or ANOVA in PRISM. The *p* values <.0001(****), <.001(***), <0.01 (**) and <0.05 (*) were considered statistically significant.

### Public RNA Seq Data Collection

All data analyzed in this article were publicly available through online sources. The raw data for the ENCODE dataset can be accessed at the ENCODE Data Coordination Center (https://www.encodeproject.org).^[^ ^25^ ^]^ The TCGA expression datasets were downloaded and processed from Broad GDAC Firehose (https://gdac.broadinstitute.org/). The TCGA splicing datasets were downloaded and processed from ASCancer Altas^[^ ^71^ ^]^ (https://ngdc.cncb.ac.cn/ascancer/home). The RNA‐seq dataset of the 50 paired hepatocellular carcinoma patients can be accessed with GSE77314.^[^ ^34^ ^]^ The RNA‐seq dataset of human mammary primary tumors ER+ and paired adjacent healthy tissues can be accessed with GSE103001.^[^ ^72^ ^]^ The RNA‐seq dataset of human primary breast cancer tumors and distant metastasis tumors can be accessed with GSE191230.^[^ ^40^ ^]^ The RNA‐seq dataset of glioblastoma tumors can be accessed in EGA with accession code EGAS00001002515.^[^ ^46^ ^]^ The single‐cell RNA‐seq dataset of 576 individual cells from the kidneys of E18.5 mouse embryos can be accessed with GSE146988.^[^ ^56^ ^]^ The single‐cell RNA‐seq dataset of primary breast cancer can be accessed with GSE75688.^[^ ^59^ ^]^ The single‐cell RNA‐seq dataset of human iPSCs and MNs can be accessed with GSE85908.^[^ ^63^ ^]^ The RNA‐seq dataset of 8 Alzheimer's disease samples and 10 non‐Alzheimer's disease samples can be accessed with GSE173955.

### Software Availability

The open‐source MRAS R package and tutorial are available at GitHub (https://github.com/zhou‐lei5/MRAS).

## Conflict of Interest

The authors declare no conflict of interest.

## Author Contributions

L.Z. and Y.H. contributed equally to this work. Z.L. conceived and supervised the entire project. L.Z. and Y.H. implemented the algorithm and performed the analyses with the help of X.W., R.X., X.L., and S.W. Y.Z. performed experiments. L.Z. and Y.H. wrote the manuscript with feedback from all other authors. All authors read and approved the final manuscript.

## Supporting information



Supporting Information

Supplemental Tables

## Data Availability

Data sharing is not applicable to this article as no new data were created or analyzed in this study.
