# Supplementary material for: MRAS: Master Regulator Analysis of Alternative Splicing
Source: Adv Sci (Weinh). 2025 May 5;12(21):2414493. doi: 10.1002/advs.202414493 (PMC12140307; doi:10.1002/advs.202414493)
Supplement: Supplementary file 1 — Supporting Information [file ADVS-12-2414493-s002.docx]

Supporting Information for

**MRAS: Master Regulator analysis of Alternative Splicing**

Lei Zhou^1,2,3^*, Yue Huang^1,2,3^*, Yang Zhao^1,2^, Dan Guo^1,2^, Xiao Wen^1,2^, Ruihong Xu^1,2,3^, Xuan Lv^1,2,3^, Song Wu^2,3,4^, Sicheng Jing^5^, Zhaoqi Liu^1,2,3#^

^1^China National Center for Bioinformation, Beijing 100101, China

^2^Beijing Institute of Genomics, Chinese Academy of Sciences, Beijing 100101, China

^3^University of Chinese Academy of Sciences, Beijing 100049, China

^4^National Genomics Data Center, China National Center for Bioinformation, Beijing, China

^5^Department of Biology, University of California San Diego, San Diego 92122, CA, USA

*These authors contributed equally

^#^Correspondence: [liuzq@big.ac.cn](mailto:liuzq@big.ac.cn) (Z.L.)


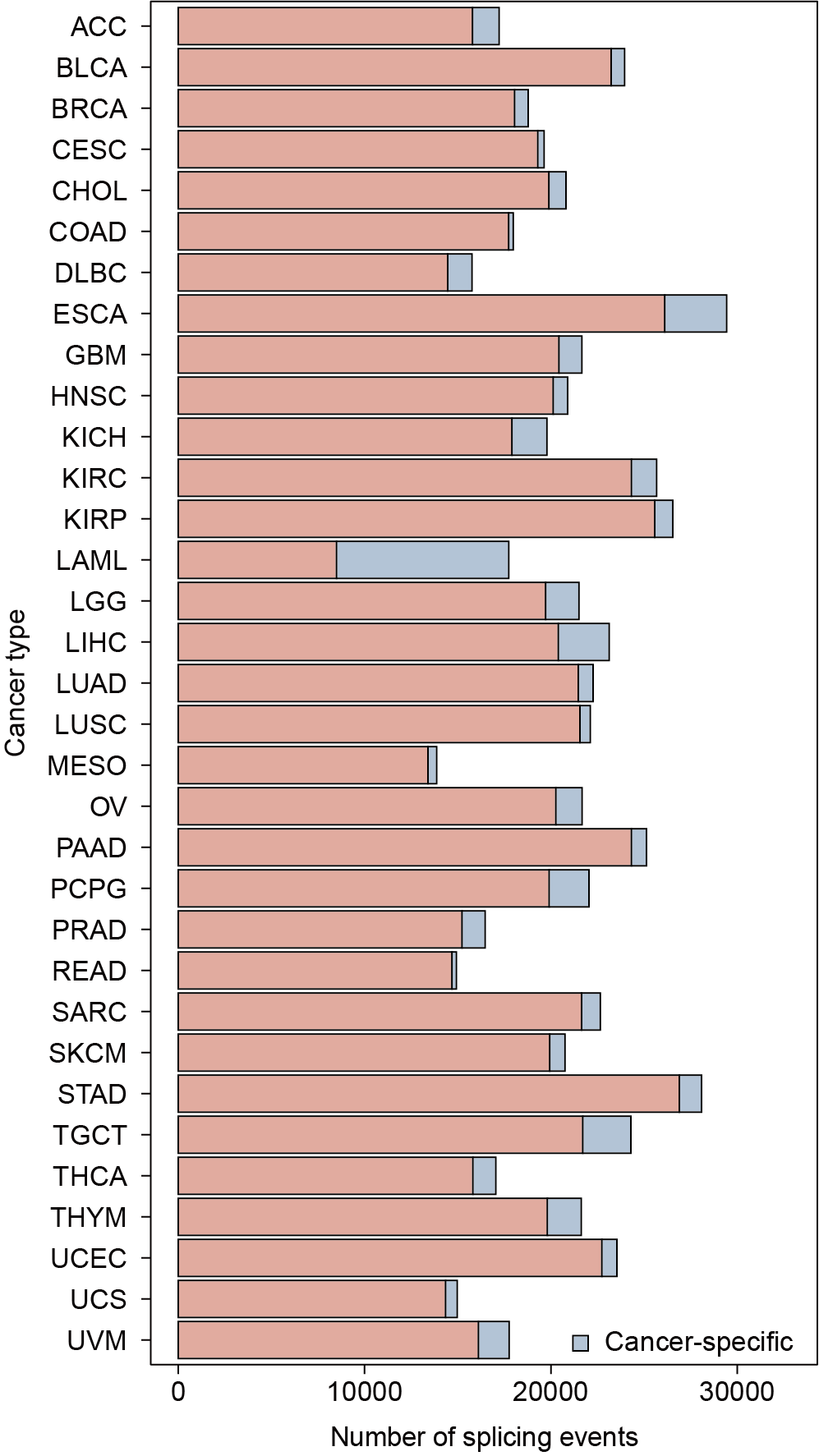


**Fig. S1. Constructed MRAS networks for 33 cancer types.** Number of splicing events in the 33 networks by MRAS. The blue bar represented cancer-specific events. ACC, Adrenocortical Cancer; BLCA, Bladder Cancer; BRCA, Breast Cancer; CESC, Cervical Cancer; CHOL, Bile Duct Cancer; COAD, Colon Cancer; DLBC, Large B-cell Lymphoma; ESCA, Esophageal Cancer; GBM, Glioblastoma; HNSC, Head and Neck Cancer; KICH, Kidney Chromophobe; KIRC, Kidney Clear Cell Carcinoma; KIRP, Kidney Papillary Cell Carcinoma; LAML, Acute Myeloid Leukemia; LGG, Lower Grade Glioma; LIHC, Liver Cancer; LUAD, Lung Adenocarcinoma; LUSC, Lung Squamous Cell Carcinoma; MESO, Mesothelioma; OV, Ovarian Cancer; PAAD, Pancreatic Cancer; PCPG, Pheochromocytoma and Paraganglioma; PRAD, Prostate Cancer; READ, Rectal Cancer; SARC, Sarcoma; SKCM, Melanoma; STAD, Stomach Cancer; THCA, Thyroid Cancer; THYM, Thymoma; TGCT, Testicular Cancer; UCS, Uterine Carcinosarcoma; UCEC, Endometrioid Cancer; UVM, Ocular melanomas.

**
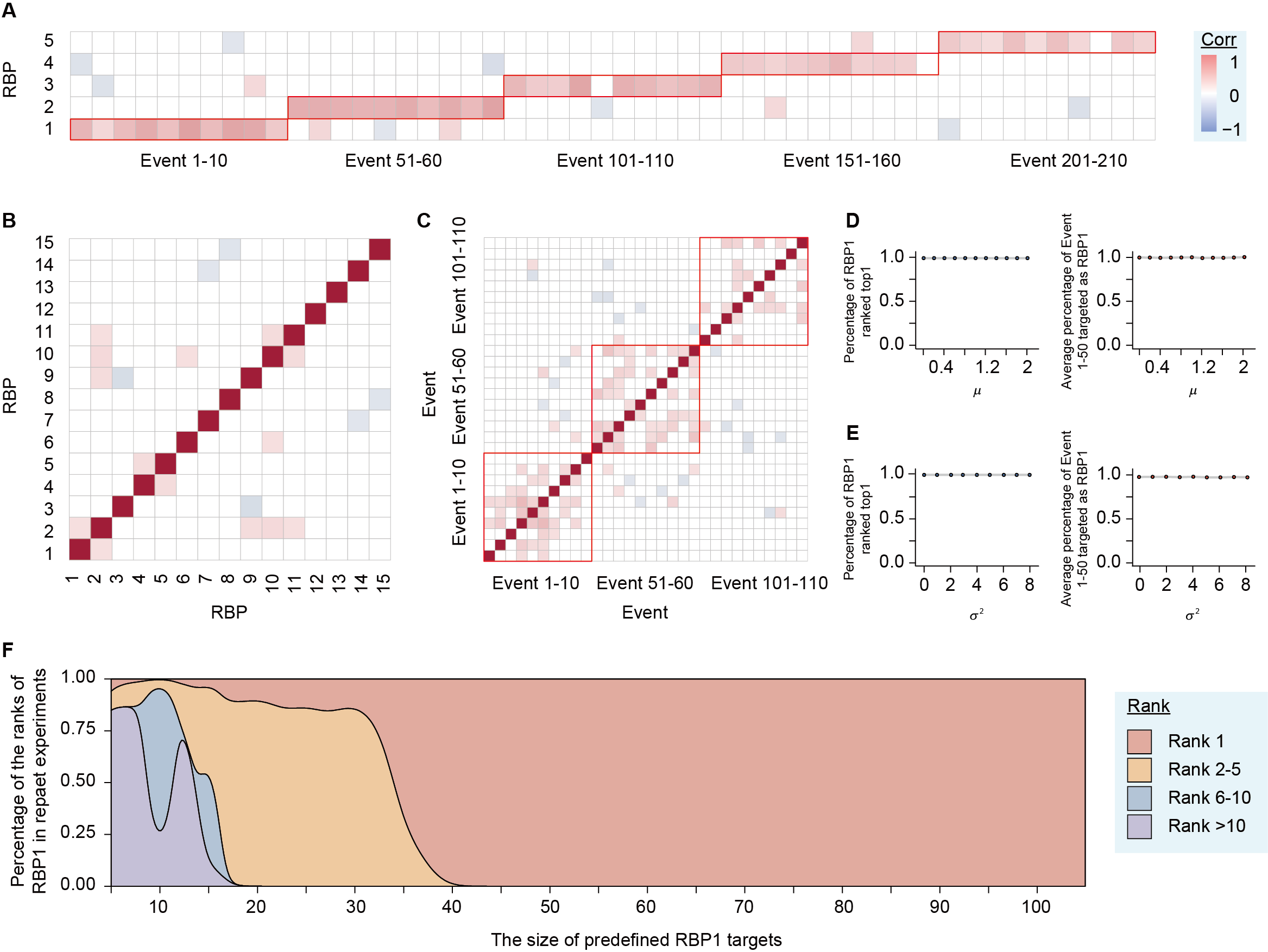
**

**Fig. S2. Predefined RBP-event correlations and parameter test in simulation studies. A**) Correlations of 10 predefined splicing events for RBP1-5. Here, events 1~10 were predefined as targets of RBP1, events 51~60 of RBP2, events 101~110 of RBP3, events 151~160 of RBP4, and events 201~210 of RBP5. **B**) Expression correlations between RBP1~15. **C**) Correlations between predefined splicing events of RBP1-3. Here, events 1~10 were predefined targets of RBP1, events 51~60 of RBP2, and events 101~110 of RBP3. **D**) (left) Percentage of RBP1 identified as the top one regulator by MRAS in 100 repeats at each $\mu$ value. (right) Percentage of events 1 to 50 inferred as RBP1 targets by MRAS in 100 repeats at each $\mu$ value. **E**) (left) Percentage of RBP1 identified as the top one regulator by MRAS in 100 repeats at each $\sigma^{2}$ value. (right) Percentage of events 1 to 50 inferred as RBP1 targets by MRAS in 100 repeats at each $\sigma^{2}$ value. **F**) Percentage of the ranks of RBP1 in 100 repeats (y-axis) at different sizes of predefined RBP1 targets (x-axis).


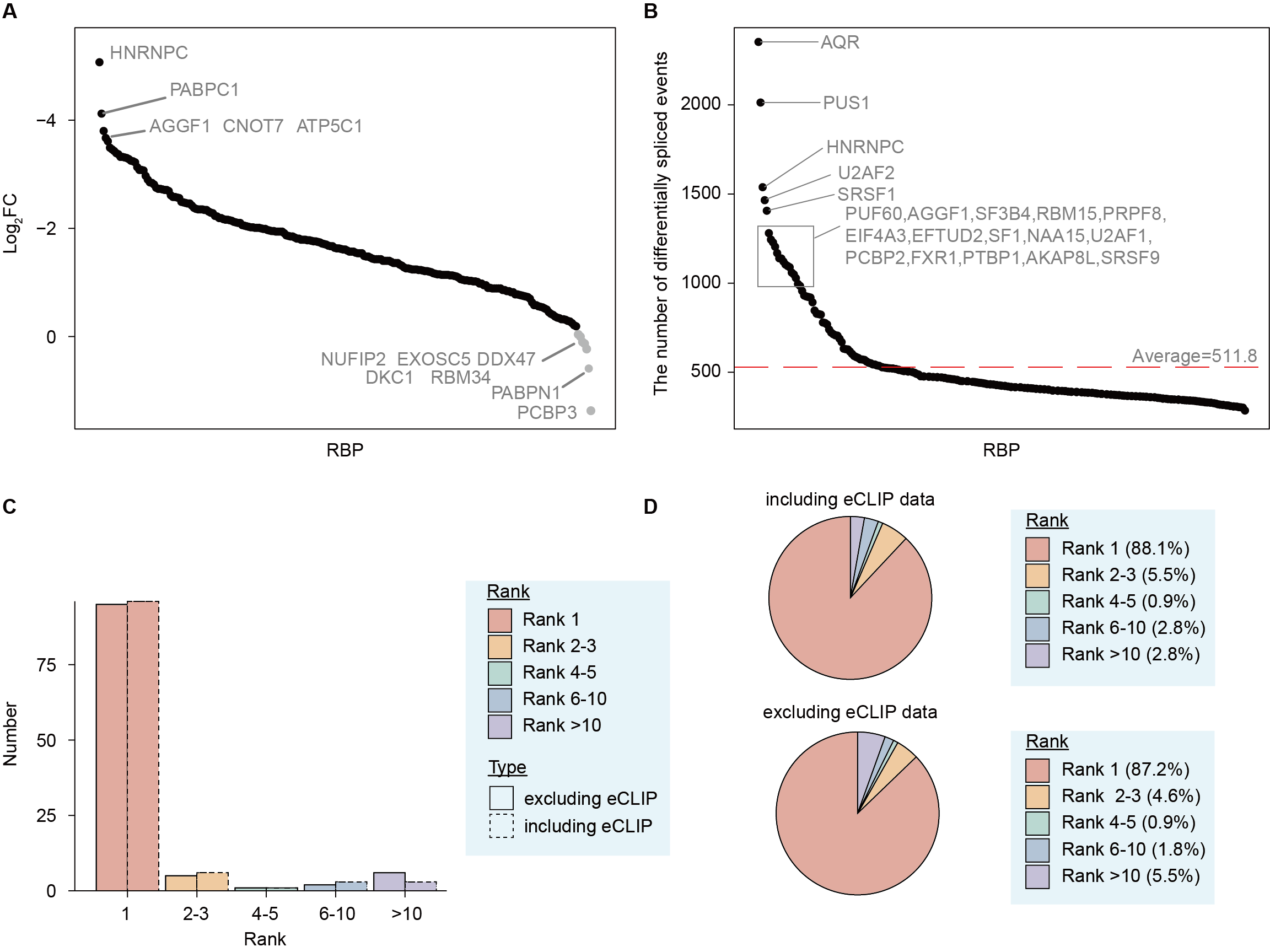


**Fig. S3. MRAS performance testing on ENCODE RNASeq datasets of RBP knockdown experiments. A**) Ranking of 235 RBPs based on log2 fold change of RBP expression between knockdown and control. The 7 grey ones were excluded from the analysis. **B**) The number of differentially spliced events number under each RBP knockdown. The red line indicates the average number across all 228 RBPs. **C**) Counts of target RBPs that were successfully identified as top regulators by including or excluding eCLIP-seq data. **D**) Pieplot shows the distribution of different ranks by including and excluding eCLIP-seq data.


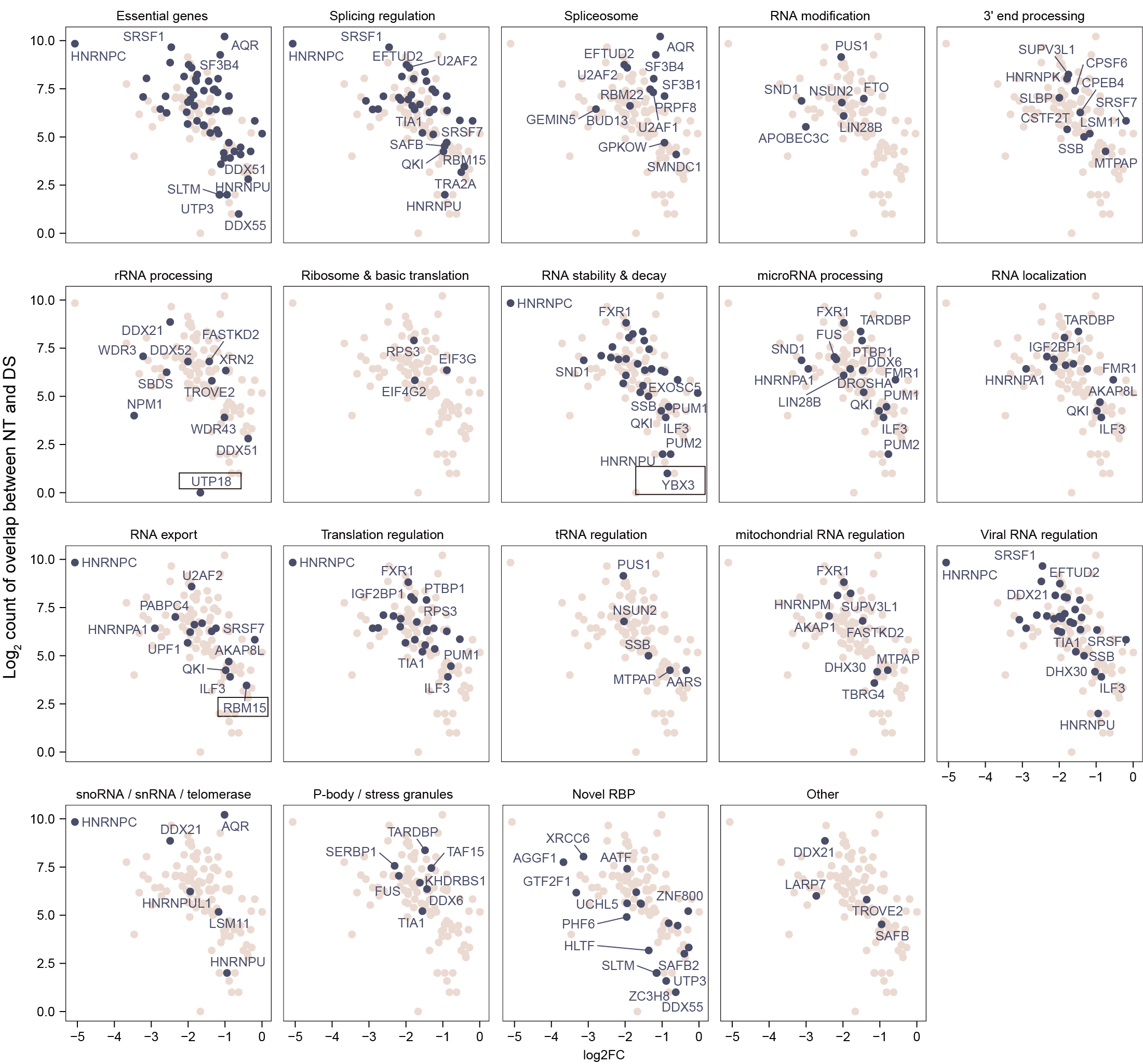


**Fig. S4. Scatterplots of RBPs with different functional annotations defined from previous study.** The figure layout is the same as Fig. 3c. Black points from each subfigure indicate RBPs associated with different functional annotations, which were directly derived from the previous ENCODE study(*25*).


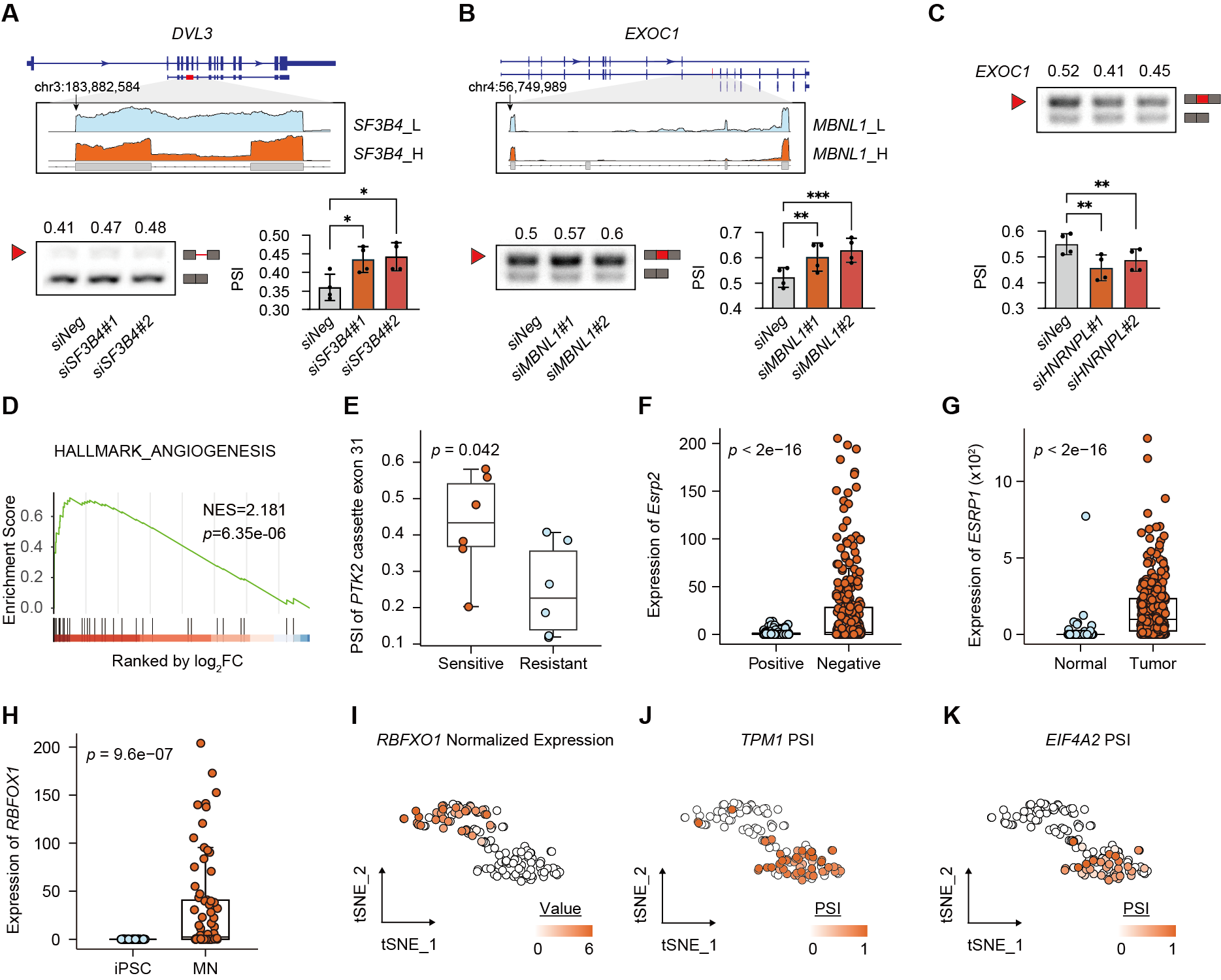


**Fig. S5. Application of MRAS in bulk RNA-seq and single-cell RNA-seq data. A**) RT-PCR was performed to validate AS changes of *DVL3* by *SF3B4* knockdown in Huh7 cells. **B**) RT-PCR was performed to validate AS changes of *EXOC1* by *MBNL1* knockdown in T47D cells. **C**) RT-PCR was performed to validate AS changes of *EXOC1* by *HNPNPL* knockdown in T47D cells. **D**) GESA enrichment of angiogenesis pathway between TCGA GBM patients with low/high 25% of PSI changes of *PTK2* cassette exon 31. PSI value indicates the inclusion level of *PTK2* exon 31. **E**) Comparison of PSI values of *PTK2* cassette exon 31 between Foretinib sensitive (*n*=6) and resistant (*n*=6) GBM patient-derived cells. PSI value indicates the inclusion level of *PTK2* exon 31. **F**) Increased expression of *Esrp2* in six2 negative cells. **G**) Increased expression of *ESRP1* in breast tumor cells. **H**) Increased expression of *RBFOX1* in MN cells. **I**) t-SNE plot with cells colored by the expression of *RBFXO1.* **J**) t-SNE plot with cells colored by PSI of *TPM1.* **K**) t-SNE plot with cells colored by PSI of *EIF4A2.* A), B), and C) PSI was quantified and the bar plot showed repeated experiment (*n* = 4).


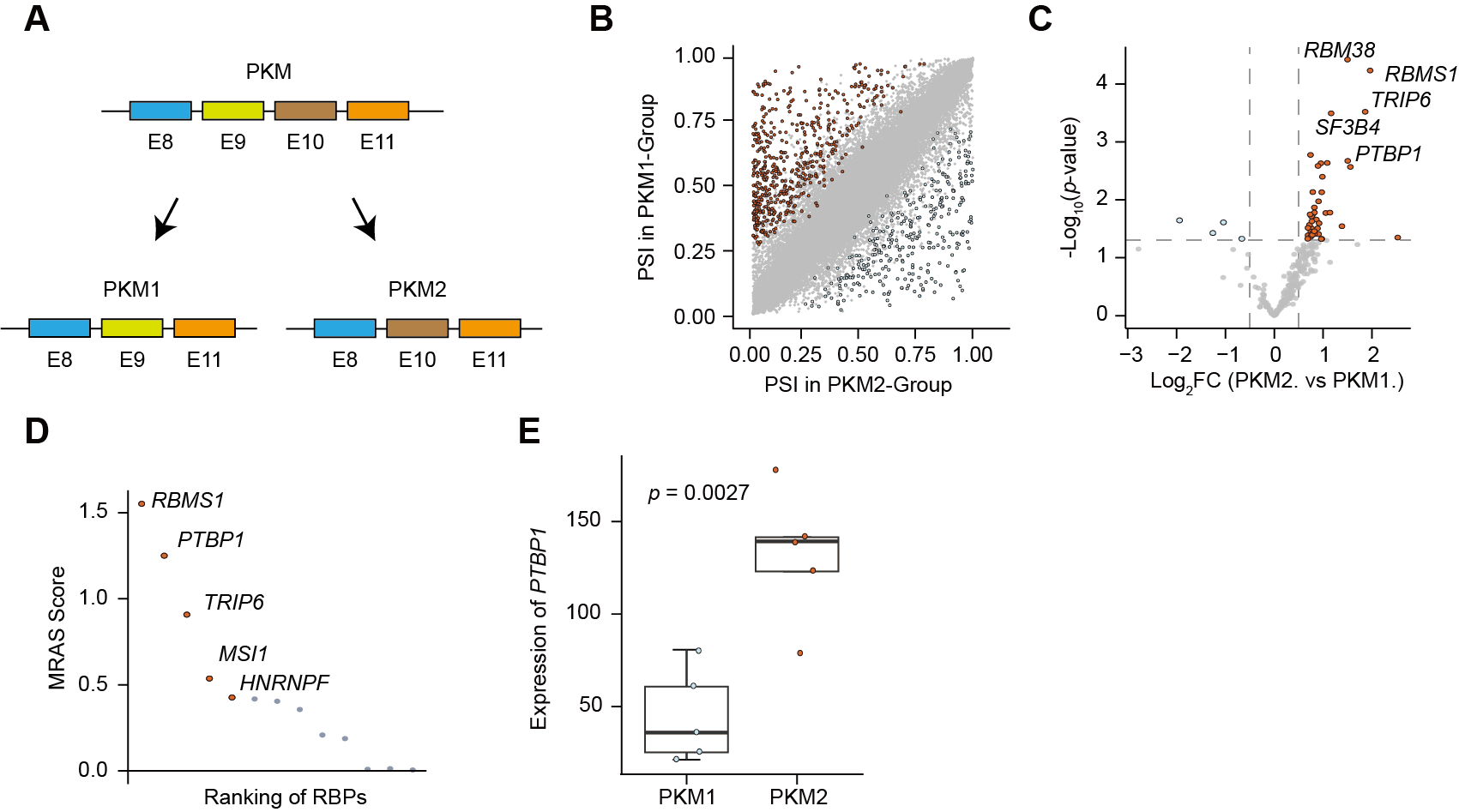


**Fig. S6. An application of MRAS on GBM samples. A**) Schema of MRAS application on PKM1-group vs PKM2-group samples. **B, C**) Differentially spliced events (events with |dPSI|>0.2 are colored) and differentially expressed RBPs between PKM1-group and PKM2-group GBM samples. **D**) A ranking of splicing regulators by MRAS predictions with the top five colored red. **E**) Increased expression of *PTBP1* in PKM2-group samples.


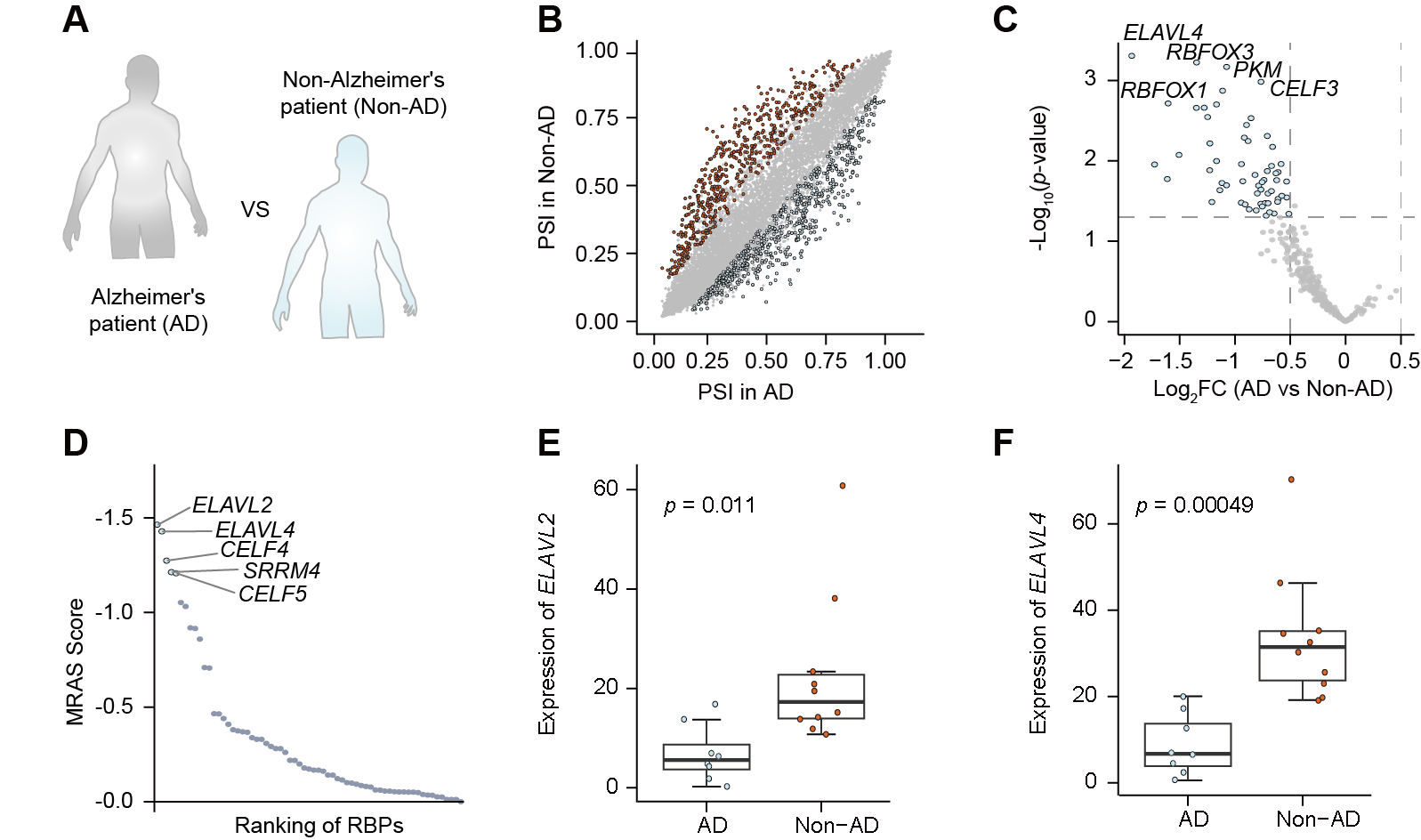


**Fig. S7. An application of MRAS on AD and non-AD samples. a**) Schema of MRAS application on AD vs non-AD samples. **b, c**) Differentially spliced events (events with |dPSI|>0.1 are colored) and differentially expressed RBPs between AD and non-AD samples. **d**) A ranking of splicing regulators by MRAS predictions with the top five colored blue. **e**) Decreased expression of *ELAVL2* in AD samples. **f**) Decreased expression of *ELAVL4* in AD samples.
